# Divergent functional outcomes of NLRP3 blockade downstream of multi-inflammasome activation: therapeutic implications for ALS

**DOI:** 10.3389/fimmu.2023.1190219

**Published:** 2023-07-27

**Authors:** Marie-Laure Clénet, James Keaney, Gaëlle Gillet, Jorge S. Valadas, Julie Langlois, Alvaro Cardenas, Julien Gasser, Irena Kadiu

**Affiliations:** ^1^ Neuroinflammation Focus Area, Neuroscience Research, Early Solutions, UCB Biopharma SRL, Braine l’Alleud, Belgium; ^2^ Development Science, Early Solutions, UCB Biopharma SRL, Braine l’Alleud, Belgium

**Keywords:** NLRP1, NLRP3, NLRC4, MEFV, inflammasomes, iPSC-derived microglia, ALS, MCC950

## Abstract

NOD-Like Receptor Family Pyrin Domain Containing 3 (NLRP3) inflammasome modulation has emerged as a potential therapeutic approach targeting inflammation amplified by pyroptotic innate immune cell death. In diseases characterized by non-cell autonomous neurodegeneration including amyotrophic lateral sclerosis (ALS), the activation of several inflammasomes has been reported. Since functional redundancy can exist among inflammasome pathways, here we investigate the effects of NLRP3 inhibition on NLRP3, NLR family CARD Domain Containing 4 (NLRC4) and non-canonical pathways to understand whether NLRP3 blockade alone can mitigate pro-inflammatory cytokine release and pyroptotic cell death in contexts where single or multiple inflammasome pathways independent of NLRP3 are activated. In this study we do not limit our insights into inflammasome biology by solely relying on the THP-1 monocytic line under the LPS/nigericin-mediated NLRP3 pathway activation paradigm. We assess therapeutic potential and limitations of NLRP3 inhibition in multi-inflammasome activation contexts utilizing various human cellular systems including cell lines expressing gain of function (GoF) mutations for several inflammasomes, primary human monocytes, macrophages, healthy and Amyotrophic Lateral Sclerosis (ALS) patient induced pluripotent stem cells (iPSC)-derived microglia (iMGL) stimulated for canonical and non-canonical inflammasome pathways. We demonstrate that NLRP3 inhibition can modulate the NLRC4 and non-canonical inflammasome pathways; however, these effects differ between immortalized, human primary innate immune cells, and iMGL. We extend our investigation in more complex systems characterized by activation of multiple inflammasomes such as the SOD1^G93A^ mouse model. Through deep immune phenotyping by single-cell mass cytometry we demonstrate that acute NLRP3 inhibition does not ameliorate spinal cord inflammation in this model. Taken together, our data suggests that NLRP3 inhibition alone may not be sufficient to address dynamic and complex neuroinflammatory pathobiological mechanisms including dysregulation of multiple inflammasome pathways in neurodegenerative disease such as ALS.

## Introduction

ALS is a debilitating and fatal neurodegenerative disorder characterized by loss of motor neurons, progressive muscle atrophy and paralysis. Among the proposed non-cell autonomous mechanisms contributing to ALS disease progression, aberrant innate immune functions in the central nervous system (CNS) (i.e., motor cortex, spinal cord) (1–8) and peripheral nervous system (PNS; i.e., neuromuscular junctions, skeletal muscle) (9–12) are emerging as an important modifiers of motor neuron health and survival. Activation of microglia and macrophages, innate immune cells of the CNS and peripheral tissues, respectively, and subsequent release of inflammatory factors are central events in the neuroinflammatory cascade. Such activation is mediated by pattern recognition receptors (PRRs) which sense molecules released during tissue damage including pathogen-associated molecular patterns (PAMPs) and danger-associated molecular patterns (DAMPs) (13). Among the PRRs receiving much attention in recent years, nucleotide-binding oligomerization domain and leucine-rich repeat-containing receptors (NLRs) form intracellular multiprotein complexes known as inflammasomes in response to PAMP/DAMP sensing (14). Inflammasome assembly results in recruitment and activation of caspase-1 which in turn cleaves inactive interleukin 1 (IL-1) family precursors such as pro-interleukin 1 beta (IL-1β) and pro-interleukin 18 (IL-18) into mature cytokines and gasdermin D facilitating formation of cell membrane pores that result in cytokine release in their transient form and more permanently in a pro-inflammatory form of cell death known as pyroptosis (15). NLRP3 is the most studied inflammasome of the twenty-member family in the context of CNS disease (16) and there is increasing evidence for its involvement in ALS pathophysiology. Firstly, levels of NLRP3 and its adaptor molecule apoptosis-associated speck-like protein (ASC) as well as active caspase-1, IL-1β and IL-18 are elevated in the spinal cord, motor cortex and/or skeletal muscle of ALS patients and in the mutant superoxide dismutase 1 (SOD1) mouse model of ALS (17–21). Secondly, it has been shown that the hallmark mutant protein aggregates including those composed of SOD1 or transactive response DNA-binding protein-43 (TDP-43) can activate the NLRP3 inflammasome in microglia (22–24) with TDP-43-mediated microglia activation causing increased motor neuron death (24). Third, a genetic knockout (KO) of caspase-1 attenuates inflammatory pathology and extends lifespan in mutant SOD1 mice (22). However preclinical and clinical data shows that other inflammasomes are dysregulated in ALS. Levels of the NLRC4 inflammasome are elevated in denervated skeletal muscle of both ALS patients and mutant SOD1 mice (20). In addition, levels of active human caspase-4 and its mouse ortholog caspase-11, central components of the non-canonical inflammasome pathway, are increased in ALS patient brains and in mutant SOD1 mouse spinal cord, respectively (25, 26). While selective inflammasome inhibition is a promising therapeutic approach in monogenic autoinflammatory syndromes associated with NLRP3- or NLRC4-activating mutations (27), we hypothesize that strategies that impact multiple inflammasome pathways may hold more promise in CNS disorders like ALS where multiple inflammasomes are dysregulated (28). Interestingly, recent evidence suggests that depending on species (29, 30), cell type (31) and inflammasome activator (30–36), functional redundancy may exist among inflammasomes suggesting that pharmacological inhibition of NLRP3 may modulate to some degree multiple inflammasome pathways. To date, non-NLRP3 inflammasome activity and the effects of NLRP3 inhibition on non-NLRP3 inflammasome pathways have not been well explored in human microglia. Furthermore, because the application of small molecule inflammasome inhibitors for ALS or other CNS indications involves systemic dosing, it is important to understand the impact of inflammasome inhibition on both peripheral and CNS resident innate immune cells. Here we investigate the impact of NLRP3 blockade in cellular models of multi-inflammasome activation including THP-1 lines carrying GoF mutations for various inflammasomes, peripheral and CNS innate immune cells including iMGL stimulated for NLRP3, NLRC4 canonical and non-canonical pathways. We extend this work to assess the acute impact of NLRP3 inhibition on CNS inflammation in a more complex system such as the mutant SOD1^G93A^ model. Regardless of its limited translatability to broader patient populations, including other familial forms or sporadic ALS, this model captures some degree of inflammatory pathway complexity including multi-inflammasome activation and as such was used in this study in combination with human cellular systems to answer a key scientific question: In a system where multiple inflammasome pathways are active, is inhibition of NLRP3 sufficient to attenuate CNS inflammation?

## Materials and methods

### Reagents

NLRP3 inhibitor tool compound MCC950 (CP-456773 sodium salt, PZ0280) and dimethyl sulfoxide (DMSO) were obtained from Sigma-Aldrich. Lipopolysaccharide (LPS) from *Escherichia coli* (O55:B5; ALX-581-013-L002) was obtained from Enzo. Nigericin (tlrl-nig-5) and ultrapure flagellins from *Bacillus subtilis* (Fla-BS; tlrl-pbsfla), *Pseudomonas aeruginosa* (Fla-PA; tlrl-pafla) and *Salmonella typhimurium* (Fla-ST; tlrl-epstfla-5) were obtained from Invivogen (purity >95% and endotoxin levels of <0.05 EU/µg). Propidium iodide (PI; P3566) and Lipofectamine 2000 (LF2000; 11668030) were obtained from ThermoFisher. Anti-mouse antibodies for mass cytometry were obtained either pre-conjugated to metal isotope or conjugated in-house using the Maxpar Antibody Labeling Kit (Fluidigm) following manufacturer’s instructions.

### Cell culture

### THP-1 cell lines

Wild-type (WT), NLRP3 knockout (KO) and familial Mediterranean fever (MEFV) gene KO THP-1 cell lines were supplied by Invivogen and WT, NLRC4 knock-in (KI), NLRP1 KI and MEFV KI THP-1 cell lines carrying GoF mutations were generated at Synthego as described in **Table S1**. THP-1 cell lines were maintained in Roswell Park Memorial Institute (RPMI-1640) medium (ThermoFisher) supplemented with 10% heat-inactivated foetal bovine serum (FBS; ThermoFisher), 1% penicillin/streptomycin (P/S; ThermoFisher) and selective antibiotics according to manufacturer’s instruction at 37°C in 5% CO_2_. THP-1 lines were differentiated into macrophage-like cells following exposure to phorbol 12-myristate 13-acetate (PMA; 100 ng/ml; Sigma-Aldrich) for 72 h and used in inflammasome activation assays.

#### Culturing and maintenance of hMonocytes and hMDM

Human monocytes (hMonocytes) isolated by negative selection from peripheral blood mononuclear cells of healthy donors were obtained under informed consent from StemCell Technologies. To generate human monocyte-derived macrophages (hMDM), monocytes were cultured for 7 days with half-medium exchanges in Dulbecco’s Modified Eagle Medium (DMEM) GlutaMAX (ThermoFisher) supplemented with 10% endotoxin-low heat-inactivated FBS, 1% P/S and human macrophage colony stimulating factor (M-CSF, 40 ng/ml, ThermoFisher) at 37°C in 5% CO_2_.

#### Generation and differentiation to iMGL

**Table S2** includes iPSC lines obtained under informed consent from Cedars-Sinai iPSC Core. All lines were assessed using the following quality control parameters: expression of pluripotency markers, functional pluripotency, karyotype analysis and STR profiling. Generation of iMGL was performed as previously described (37) with modifications. Briefly, iPSCs were first differentiated in induced hematopoietic progenitor cells (iHPC) for 10-18 days. On the first 2 days, embryoid bodies are cultured with fibroblast growth factor 2 (FGF2), bone morphogenetic protein 4 (BMP4), and activin A under hypoxic conditions. Between day 2 and 4 culture was maintained in hypoxic conditions in medium supplemented with FGF2 and vascular endothelial growth factor (VEGF). After day 4, cells were grown in normoxic conditions and medium changes were performed every 2 days with media supplemented with FGF2, VEGF, thrombopoietin (TPO), M-CSF, Interleukin 3 (IL-3), and interleukin 6 (IL-6). iHPC were differentiated in iMGL for 15 d with media supplemented with insulin, M-CSF, tumor growth factor beta 1 (TGF-ß1), and interleukin 34 (IL-34). All compounds and media were cell culture grade and purchased from ThermoFisher Scientific, Sigma, and Peprotech.

### Inflammasome activation assays

For cytokine release assays, THP-1 and hMonocytes were seeded at 5 x 10^4^ cells/well (96-well plates) or 1.6 x 10^4^ cells/well (384-well plates); hMDM and iMGL were seeded at 1 x 10^4^ cells/well (384-well plates). For PI uptake THP-1 and hMonocytes were seeded at 3.5 x 10^4^ cells/well (96-well plates) or 1.6 x 10^4^ cells/well (384-well plates); hMDM were seeded at 0.2 x 10^4^ cells/well (384-well plates); and iMGL were seeded at 1 x 10^4^ cells/well (384-well plates). MCC950 (dose response range 0.01 -50 µM) or vehicle control (DMSO 0.5% v/v) were added prior to nigericin addition (for NLRP3 activation), flagellin transfection (NLRC4) or LPS transfection (non-canonical). For NLRP3 inflammasome activation, cells were stimulated with LPS (0.1 μg/ml for hMonocytes; 1 μg/ml for THP-1 and iMGL) for 3 h followed by media exchange with nigericin (10 μM). For cytokine measurements, culture fluids were collected after 1 h (hMonocytes, hMDM), 2 h (THP-1) or 3 h (iMGL), centrifuged at 500 *xg* for 5 min to remove cell debris and stored at -80°C until further use. For cell death imaging assays, PI (0.5-1 μg/ml) was added after nigericin addition and membrane permeability was measured longitudinally through time lapse imaging, normalized for confluency to account for either cell proliferation or cell death at 37° C and 5% CO_2_ using a cell culture incubator housed IncuCyte (Sartorius). For NLRC4 inflammasome activation, cells were stimulated with flagellin from *Bacillus subtilis* (Fla-BS), *Pseudomonas aeruginosa* (Fla-PA) or *Salmonella typhimurium* (Fla-ST; 100 ng/well for THP-1, 50-500 ng/well for hMDM, 500 ng/well for iMGL) transfected with LF2000 (0.25-2.5 μl/well). For cytokine measurements, supernatants were collected after 24 h. PI uptake was measured following addition to the cell cultures at a concentration of 1 μg/ml post- flagellin transfection.

#### Non-canonical inflammasome activation

For non-canonical inflammasome activation, THP-1 lines were pre-incubated with Pam3CysSerLys4 (PAM3CSK4) (1 μg/ml) for 3 h followed by transfection with LPS (100 ng/well) using LF2000 (0.5 μl/well). For cytokine measurements, culture fluids collected after 24 h were centrifuged to remove cellular debris and stored at -80° C. PI uptake was assessed following LPS transfection as described above.

### Cytokine measurements

Supernatants from THP-1 lines, hMonocytes and hMDM were tested for interleukin 1 alpha (IL-1α), IL-1β, IL-18 and tumor necrosis factor alpha (TNF-α) levels using the relevant human Cisbio homogeneous time resolved fluorescence (HTRF) kits following manufacturer’s instructions. Supernatants from iMGL were tested for IL-1β levels using the Meso Scale Discovery (MSD) V-Plex assay kit following manufacturer’s instructions.

### Estimation of MCC950 target occupancy *in vivo*

The pharmacokinetics (PK) profiles of MCC950 at the dose of 20 mg/kg in mice were extracted from the plasma concentration-time graphs reported by Gordon et al. [58] and Coll et al. [44] using Digitizelt software (Version 1.5.8; Braunschweig, Germany). Plasma concentrations were fitted to a mono-compartmental PK model using Phoenix WinNonlin (v8.3, St Louis, MO). Estimates of free plasma concentration-time profile were obtained from the PK model, correcting total plasma concentration by multiplying with the obtained *in vitro* fraction unbound in plasma (**Table S3**). Estimates of free brain concentration were obtained by multiplying the free plasma concentration with the unbound partition coefficient (K_p,uu_), brain value observed in the blood-brain barrier mouse study. Free concentrations were expressed in nM, under the assumption that PK in SOD1^G93A^ transgenic mice was equivalent to PK in C57Bl/6 mice and it did not change during the duration of the study. Target occupancy (TO) was estimated by the equation %TO=[MCC950]*100/([MCC950] + inhibitory concentration by 50% (IC_50_; **Table S4**).

### *In vivo* dosing of MCC950

SOD1^G93A^ transgenic (B6SJL-Tg(SOD1*G93A)1Gur/J, Jackson Lab Stock no. 002726) and WT (non-carrier) mice were in-licensed from Northwestern (Northwestern University, Evanston, Illinois 60208, US) and accessed through Charles River (Charles River Laboratories, France). Mice (males only) were maintained on a 12/12-hour light/dark cycle with lights on at 06:00 h and had ad libitum access to food and drinking water. The temperature in the husbandry was maintained at 22°C and humidity at about 40%. Mice were administrated daily, over 7 days, with a single dose (oral gavage, 20 mg/kg) of MCC950/Vehicle (1X PBS). Twenty-four hours after the last dose, animals were anesthetized with isoflurane using insufflating masks. Following verification of complete sedation through paw and tail pinch, transcardial perfusion with 1X Hanks’ Balanced Salt Solution (HBSS) and 10 U/mL heparin was performed using a peristaltic pump at a rate of 6 ml/min for 5 mins. Brains and spinal cord were collected and processed for cytometry by time of flight (CyTOF) analysis. All experimental procedures and strain-specific humane endpoints were reviewed and approved by a local Animal Experimentation and Well-Being Ethical Committee compliant with national legislation guidelines (Belgian Royal Decree regarding the protection of laboratory animals of 29 May 2013) and the European directive (2010/63/EU).

### SOD1^G93A^ mouse tissue collection and processing for mass cytometry

Anaesthetized mice were perfused with 1X HBSS (10U/ml heparin) and forebrains, spinal cord and spleens were collected in ice-cold 1X HBSS. Brains were dissociated using the Papain Dissociation System (Worthington Biochemical) by gentle sequential trituration, before and after incubation of 30 min at 37°C and 5% CO_2_. Single cell suspensions were filtered through a 40 µm cell strainer (Falcon) and resuspended in 30% Percoll^®^ (Sigma-Aldrich) in AutoMACS running buffer at room temperature and centrifuged for 15 min at 500 *xg* at room temperature with no brake to allow for formation of a floating myelin layer. Following myelin removal, pelleted cells were resuspended in AutoMACS Running buffer (Miltenyi Biotec), filtered again, and centrifuged at 300 *xg* for 10 min to obtain an immune cell-enriched pellet. Spleens were homogenized by mechanical dissociation followed by red blood cell removal using 1X eBioscience RBC lysis buffer (ThermoFisher) for 10 min at room temperature and filtered through a 40 µm cell strainer.

### Mass cytometry data acquisition and analysis

Spinal cord, brain and spleen cell pellets were washed with ice-cold Maxpar Cell Staining Buffer (Fluidigm) and stained with a cocktail of 26 metal-tagged antibodies (1:100 in Maxpar Cell Staning Buffer, **Table S5**) for 1 h (100 µl final staining volume per sample). Cells were washed thrice in Maxpar cell staining buffer and fixed overnight in 4% paraformaldehyde (PFA) in 1X PBS at 4°C. Prior to data acquisition, Maxpar fixed cells were incubated overnight at 4°C with 191/193Ir DNA Intercalator (50 nM, Fluidigm) diluted in 4% PFA. Cells were washed in Maxpar H_2_O (Fluidigm) and centrifuged for 5 min at 800 *xg*. Cells were then re-suspended in EQ Four Element Calibration beads (Fluidigm) diluted in Maxpar H_2_O (1:4), filtered through a 40 µM cell strainer and acquired on a Helios mass cytometer (Fluidigm) with events acquired at an event rate of maximum 500 events per second. Mass cytometry data were analyzed using different computational tools available in Cytobank (https://cytobank.org). Single intact cells were gated on event length and DNA content to exclude debris and doublets. Identification of cell subsets was then performed using traditional bivariate gating or unsupervised high-dimensional visualization of t-distributed Stochastic Neighbor Embedding (viSNE) analysis on concatenated flow cytometry standard (FCS) files pooling cells from all mice of each group, using a t-distributed stochastic linear embedding (t-SNE) algorithm. Cell populations were defined based on the following gating strategy: microglia/resident myeloid (CD45^low^CD11b^+^CX3CR1^+^); neutrophils (CD45^high^CD11b^+^Ly-6G^+^); monocytes/macrophages (CD45^high^CD11b^+^Ly-6G^-^); dendritic cells (CD45^high^CD11b^+^CD11c^+^); T cells (CD45^high^CD11b^low^CD3^+^). The percentage of each cell population was calculated out of the total of CD45^+^ cells.

### Statistical analysis

Data and statistical analysis were performed utilizing GraphPad Prism 9.0 software. Results are presented as means ± standard deviation (S.D.) unless otherwise stated. Data were analyzed using two-tailed Student’s *t*-test to compare between two groups, one-way or two-way ANOVA followed by Tukey’s *post-hoc* test for multiple comparison test with a p value of less than 0.05 considered statistically significant.

## Results

### NLRP3 KO or pharmacological inhibition with MCC950 blocks NLRP3 inflammasome pathway activation in THP-1, hMonocytes, hMDM, and iMGL

**Figure 1:**
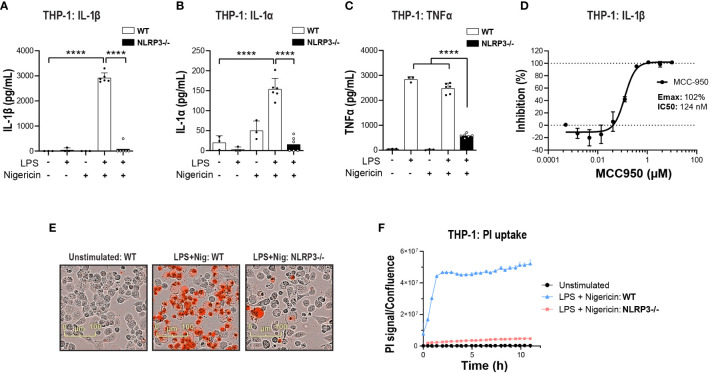
NLRP3 KO or pharmacological inhibition blocks NLRP3 pathway activation in THP-1 cells. PMA-differentiated WT or NLRP3 KO THP-1 cells were pre-incubated with LPS (1 μg/ml, 3 h) followed by nigericin (10 μM at 2 h) to activate the NLRP3 inflammasome and the release of **(A)** IL-1β, **(B)** IL-1α and **(C)** TNF-α was measured. **(D)** Dose-dependent MCC950-mediated inhibition of IL-1β release from PMA-differentiated WT THP-1 cells following LPS (1 μg/ml, 3 h) and nigericin (10 μM, 2 h) stimulation. **(E)** Representative time-lapse microscopy images of PI uptake as a measure of pyroptosis in WT and NLRP3 KO THP-1 cells stimulated with LPS and Nigericin. **(F)** Time-lapse monitoring of PI uptake in WT and NLRP3 KO THP-1 cells up to 11 h post-stimulation with LPS and Nigericin. Data are shown as mean ± S.D. of four technical replicates representing three independent experiments. Statistical analysis was performed using one-way ANOVA followed by Tukey’s *post hoc* test. ****p < 0.0001.

The search for potent and brain penetrant inhibitors of NLRP3 has become an area of drug discovery focus in recent years with biological validation relying heavily on data generated in various rodent models of neurodegenerative disease with a reference compound MCC950. The THP-1 monocytic line has been heavily used for high throughput screening (HTS) and biological investigation of NLRP3 inhibition; however, differences in inflammasome biology in the THP-1 line compared to primary human innate immune cells has not been thoroughly investigated. Beyond basic biological understanding, addressing this question may drive decision-making for deployment of functional orthogonal hit triaging earlier in the screening cascade to avoid false positive or negative outcomes. Previous studies have confirmed NLRP3 is primarily expressed in cells of the myeloid lineage in both the periphery (38) and CNS (23) and this is confirmed at the transcriptomic level using two online databases, The Human Protein Atlas (39, 40) and Brain RNA-Seq (41, 42). Therefore, to investigate the effects of NLRP3 blockade we utilized THP-1, hMonocytes, hMDM and iMGL from donors of a healthy aging cohort and ALS patients carrying various SOD1 pathogenic mutations. To activate the NLRP3 inflammasome we adopted the conventional two-hit method consisting of a priming step with LPS, a toll-like receptor 4 (TLR4) agonist to upregulate NLRP3 and pro-IL-1β and an activation step with PAMP: nigericin, to trigger NLRP3 inflammasome assembly, caspase-1 activation, and IL-1β cleavage and release (43). In THP-1 cells, KO of NLRP3 significantly decreased the release of IL-1β and another IL-1 family member, IL-1α, following LPS and nigericin stimulation (**Figures 1A, B**). TNF-a release was decreased in KO cells likely through NLRP3-independent mechanisms since LPS and nigericin stimulation induced similar TNF-α levels to LPS alone (**Figure 1C**). Attenuation of cytokine release was paralleled by decreased pyroptotic activity measured by the PI uptake (**Figures 1E, F**).

**Figure 2:**
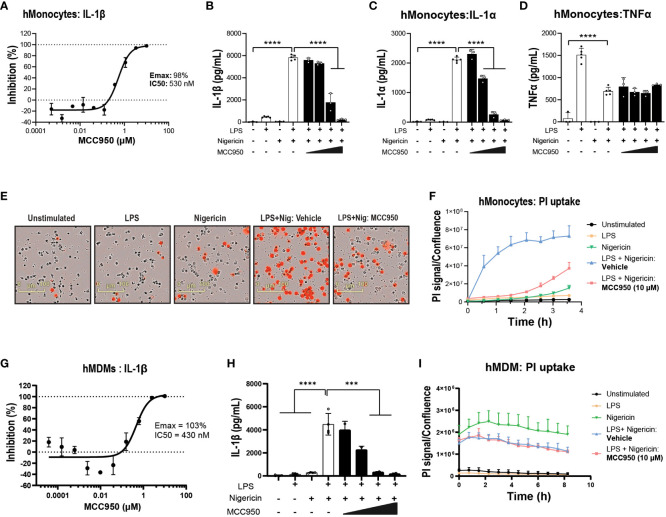
Pharmacological inhibition with MCC950 blocks NLRP3 pathway activation in hMonocytes. **(A)** MCC950 dose-dependent inhibition of IL-1β release from primary human monocytes following LPS (0.1 μg/ml, 3 h) and nigericin (10 μM, 1 h) stimulation. Effects of MCC950 (0.37-10 μM) on **(B)** IL-1β, **(C)** IL-1α and **(D)** TNF-α release from hMonocytes following LPS (0.1 μg/ml, 3 h) and nigericin (10 μM, 1 h) stimulation. **(E)** Representative images of PI uptake in hMonocytes following various treatments. **(F)** Time-lapse imaging analysis of PI uptake up to 4 h post-treatment in hMonocytes (signal normalized to cellular confluency). **(G)** Dose-dependent inhibition of IL-1β release by MCC950 (0.01-50 μM) and **(H)** at top two concentrations in the dose response curve in hMDM following LPS (0.1 μg/ml, 3 h) and nigericin (10 μM, 1 h) stimulation. **(I)** Time lapse imaging of PI uptake normalized to cellular confluency in hMDM exposed to various stimuli and MCC950 at 10 μM, over 8 (h) Data are shown as mean ± S.D. of four technical replicates from four healthy donors. One‐way ANOVA followed by Tukey’s *post hoc* test. ***p < 0.001 and ****p < 0.0001.

Next, to compare NLRP3 KO with pharmacological inhibition, the small molecule NLRP3 inhibitor MCC950 was used (44). MCC950 blocked IL-1β release following LPS and nigericin stimulation in both THP-1 (Emax: 102%, IC_50_: 124 nM) and primary hMonocytes (Emax: 98%, IC_50_: 530 nM) (**Figure 1D** and **Figures 2A, B**), with higher IC_50_ values than reported previously (44, 45). This is likely due to the presence of serum in cell assays and the reported high protein binding of MCC950 (45). In hMonocytes, increasing MCC950 concentrations resulted in a significant blockade of IL-1α release (**Figure 2C**). Interestingly, in hMonocytes LPS induced a higher release of TNFα compared to LPS + Nigericin in THP-1, and unlike in THP-1, MCC90 did not attenuate TNFα release (**Figure 2D**). MCC950 significantly reduced PI uptake in hMonocytes following LPS and nigericin stimulation (**Figures 2E, F**). A dose-dependent decrease of IL-1β release was observed with MCC950 treatment following LPS and nigericin stimulation in hMDM (Emax: 103% and IC50: 430 nM; **Figure 2G**); however, the significant decrease in IL-1β at top two concentrations (10 and 50 μM) in the dose response curve (**Figure 2H**) was not accompanied by a significant blockade of pyroptotic activity in these cells (**Figure 2I**).

**Figure 3:**
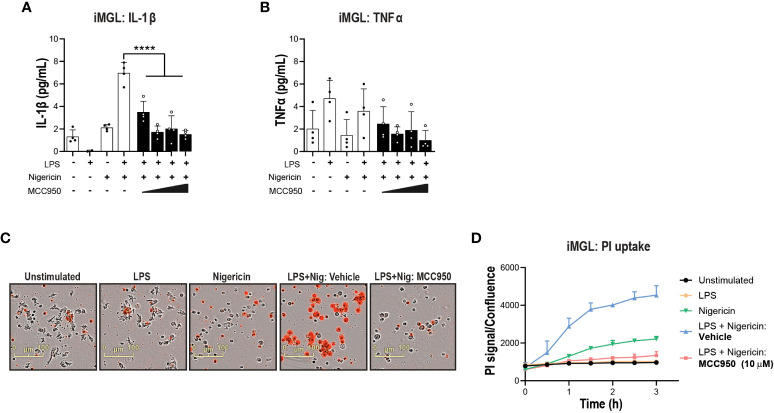
NLRP3 KO or pharmacological inhibition blocks NLRP3 pathway activation in iPSC-derived microglia. MCC950 (0.37-10 μM) dose response on **(A)** IL-1β and **(B)** TNF-α release from iMGL (line HC4) following LPS (1 μg/ml at 3 h) and nigericin (10 μM, 3 h) stimulation. **(C, D)** Effects of MCC950 on PI uptake in iMGL following LPS and nigericin stimulation. For measurements of MCC950 effects on PI uptake, dye was added directly after nigericin addition and iMGL were imaged over the following 3 h. **(C)** Representative live-cell microscopy images of PI staining of iMGL with different treatments. Data are shown as mean ± S.D. of four technical replicates from four donors. One‐way ANOVA followed by Tukey’s *post hoc* test. ****p < 0.0001.

Next, we questioned the propensity of iPSC-derived microglia to respond to NLRP3-inducing stimuli. NLRP3 activation increased IL-1β release in iMGL, albeit at lower levels compared to hMonocytes. The release of IL-1β in iMGL was significantly decreased with increasing concentrations of MCC950 (**Figure 3A**). Similar to hMonocytes, TNFα levels did not differ between LPS alone and LPS + Nigericin combination and exposure to incremental doses of MCC950 did not impact TNFα levels (**Figure 3B**). Moreover, increased PI uptake following LPS and nigericin stimulation was significantly decreased by MCC950 at the top of the dose response curve recorded for IL-1β inhibition (10µM; **Figures 3C, D**). Altogether, these data indicate that NLRP3 blockade by MCC950 result in similar responses (with the exception of TNFα release) across immortalized, primary peripheral innate immune and microglial cells following NLRP3 inflammasome activation.

### Divergent responses to MCC950 following NLRC4 inflammasome activation in THP-1 cells, hMonocytes, hMDM, and iMGL

Studies have shown that multiple inflammasomes including NLRP3, NLRC4, and apoptosis inhibitor of macrophage 1 (AIM1) among others are active in complex human CNS diseases including Alzheimer’s disease (AD), ALS, multiple sclerosis (MS) and stroke (16, 20, 46, 47). Given clinical advancement of NLRP3 inhibitors in neurodegenerative disease, we questioned whether NLRP3 inhibition is sufficient to mitigate cytokine release and pyroptosis downstream of other inflammasomes such as NLRC4.

**Figure 4:**
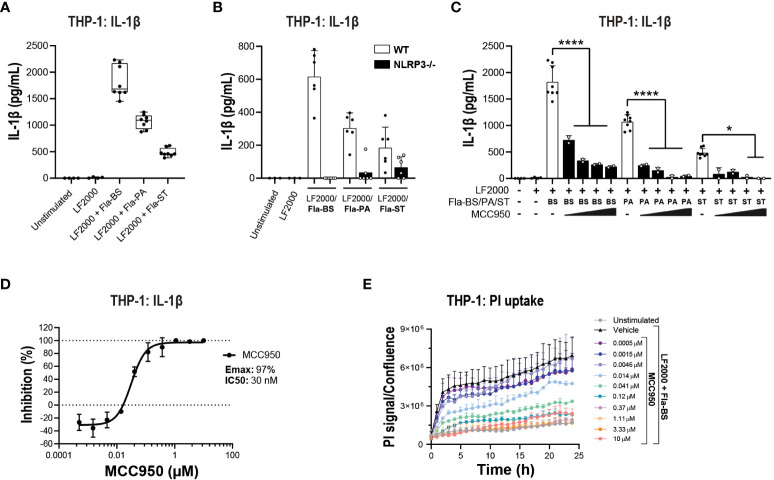
NLRP3 pharmacological inhibition blocks bacteria-induced NLRC4 pathway activation in THP-1 cells. **(A)** PMA-differentiated WT THP-1 cells and **(B)** NLRP3 KO THP-1 cells were transfected with flagellins from *Bacillus subtilis* (Fla-BS), *Pseudomonas aeruginosa* (Fla-PA) or *Salmonella typhimurium* (Fla-ST; 100 ng/well) using LF2000 (0.5 μl/well) to activate the NLRC4 inflammasome and the release of IL-1β was measured after 24 h. **(C)** Effects of MCC950 (0.37-10 μM) on IL-1β release from PMA-differentiated WT THP-1 cells following transfection with Fla-BS, -PA or -ST. **(D)** Concentration-response curve for MCC950 in blocking IL-1β release from PMA-differentiated WT THP-1 cells following transfection with Fla-BS. **(E)** Effects of MCC950 on PI uptake in PMA-differentiated WT THP-1 cells following transfection with Fla-BS and imaging over 24 h. Data are mean ± S.D. of three technical replicates and three independent experiments. One‐way ANOVA followed by Tukey’s *post hoc* test. *p < 0.05 and ****p < 0.0001.

**Figure 5:**
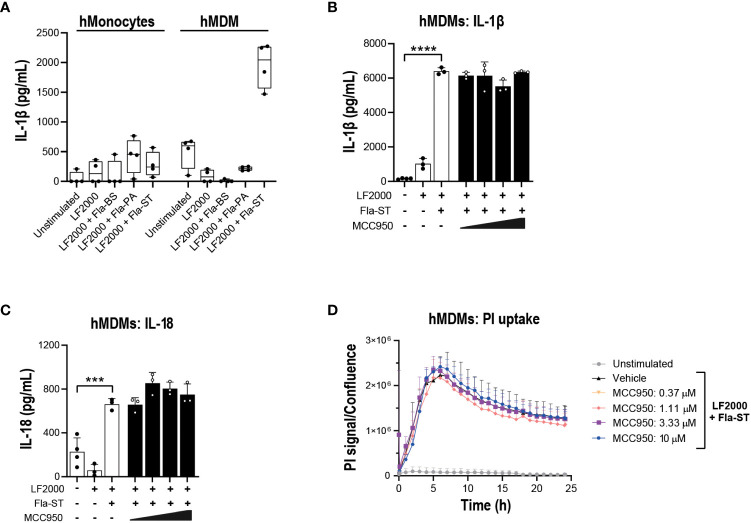
NLRP3 pharmacological inhibition does not block NLRC4 pathway activation in hMonocytes and hMDM. **(A)** hMonocytes or hMDM were transfected with flagellins from *Bacillus subtilis* (Fla-BS), *Pseudomonas aeruginosa* (Fla-PA) or *Salmonella typhimurium* (50 ng/well) using LF2000 (0.25 μl/well) and the release of IL-1β was measured after 24 h. Effect of MCC950 (0.37-10 μM) on **(B)** IL-1β and **(C)** IL-18 release from hMDM following transfection with Fla-ST (500 ng/well) using LF2000 (2.5 μl/well). **(D)** Effect of MCC950 (0.37-10 μM) on PI uptake in hMDM following transfection with Fla-ST (500 ng/well) using LF2000 (2.5 μl/well) and imaging over 24 h. Data are mean ± S.D. of three technical replicates and representative of three and four donors for hMonocytes and hMDM, respectively. One‐way ANOVA followed by Tukey’s *post hoc* test. ***p < 0.001 and ****p < 0.0001.

**Figure 6:**
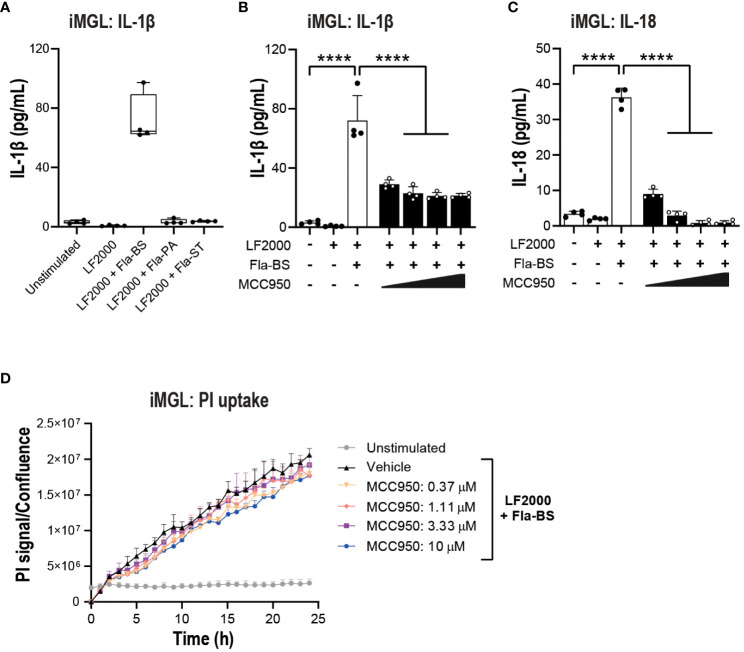
NLRP3 pharmacological inhibition blocks some aspects of NLRC4 pathway activation in iMGL. **(A)** iMGL were transfected with flagellins from *Bacillus subtilis* (Fla-BS), *Pseudomonas aeruginosa* (Fla-PA) or *Salmonella typhimurium* (500 ng/well) using LF2000 (0.25 μl/well) and the release of IL-1β was measured after 24 (h) Effect of MCC950 (0.37-10 μM) on IL-1β **(B)** and IL-18 **(C)** release from iMGL following transfection with Fla-BS (500 ng/well) using LF2000 (0.25 μl/well). **(D)** Effects of MCC950 (0.37-10 μM) on PI uptake in iMGL following transfection with Fla-BS (500 ng/well) using LF2000 (0.25 μl/well) and time-lapse imaging during 24 h. Data are mean ± S.D. of four technical replicates from four donors. One‐way ANOVA followed by Tukey’s *post hoc* test. ****p < 0.0001.

Prior to performing functional assays, we confirmed the expression of NLRC4 expression in various cell types used in the assays (39, 40). To activate the NLRC4 inflammasome, we performed cell transfections of ultrapure flagellin proteins isolated from *Bacillus subtilis* (Fla-BS), *Pseudomonas aeruginosa* (Fla-PA) or *Salmonella typhimurium* (Fla-ST) (48). In THP-1, cytosolic delivery of Fla-BS induced the strongest release of IL-1β followed by Fla-PA and Fla-ST (**Figure 4A**). Knockout of NLRP3 (**Figure 4B**) or treatment with MCC950 (**Figure 4C**) strongly decreased IL-1β release by THP-1 cells following transfection with Fla-BS, Fla-PA or Fla-ST. In addition, MCC950 was efficacious (Emax: 97%) and potent (IC_50_: 30 nM) in inhibiting IL-1β release by THP-1 following Fla-BS transfection (**Figure 4D**), and this inhibition was accompanied by a dose-dependent decrease in PI uptake (**Figure 4E**). IL-18, another cytokine released in tandem with IL-1β upon inflammasome activation has been proposed to prime the adaptive inflammatory responses in acute and chronic neurodegeneration (49). To understand the impact of NLRP3 inhibition on NLRC4-induced IL-18 release we measured IL-18 levels in THP-1 cells transfected with the different flagellins, however levels were under detectable range. Interestingly, in contrast to THP-1, hMonocytes displayed low IL-1β release following flagellin transfection regardless of bacterial source whereas hMDM responded to Fla-ST transfection only (**Figure 5A**). Based on these findings and to get closer to primary human biology, we chose to explore the effects of NLRP3 inhibition in hMDM following Fla-ST transfection. Unlike in the THP-1 line, MCC950 did not block the release of flagellin-induced IL-1β release in hMDM (**Figure 5B**). We also measured IL-18 levels in hMDM transfected with Fla-ST. As expected, IL-18 release was increased following Fla-ST transfection, however MCC950 had no effect on IL-18 levels (**Figure 5C**). PI uptake was also increased in hMDM following Fla-ST transfection; however, MCC950 treatment did not mitigate NLRC4-induced pyroptosis (**Figure 5D**). In iMGL, increased IL-1β release was observed after transfection with Fla-BS but not Fla-PA or Fla-ST (**Figure 6A**). MCC950 was able to decrease cytosolic Fla-BS-induced IL-1β release by 50-60% (**Figure 6B**) while IL-18 release was almost completely prevented with higher concentrations of MCC950 following Fla-BS transfection (**Figure 6C**). On the other hand, cytosolic Fla-BS-induced pyroptosis as measured by PI uptake was not decreased by MCC950 (**Figure 6D**). In summary, this data indicates that innate immune cells respond differently to cytosolic flagellin exposure depending on its bacterial source and that the impact of NLRP3 inhibition on NLRC4 inflammasome activation differs between immortalized and primary innate immune and iPSC-derived microglia. It also suggests that the impact of NLRP3 blockade on immune activation downstream of other inflammasomes is limited.

### MCC950 blockade attenuates some aspects of non-canonical inflammasome pathway activation

**Figure 7:**
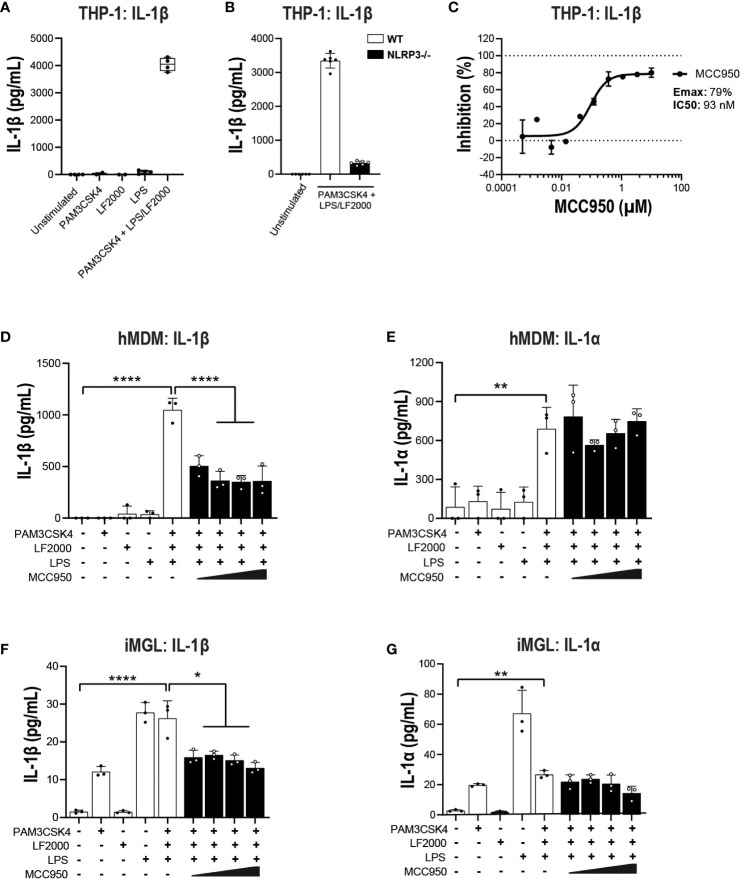
MCC950 impact on non-canonical inflammasome pathway activation in THP-1 cells and hMDM. **(A)** PMA-differentiated WT THP-1 cells and **(B)** NLRP3 KO THP-1 were pre-incubated with PAM3CSK4 (1 μg/ml, 3 h) followed by transfection with LPS (100 ng/well, 24 h) using LF2000 (0.5 μl/well) to activate the non-canonical inflammasome pathway and the release of IL-1β was measured. **(C)** Dose-dependent inhibition of IL-1β release from PMA-differentiated WT THP-1 cells following incubation with PAM3CSK4 and LPS transfection. Effect of MCC950 (0.37-10 μM) on **(D)** IL-1β and **(E)** IL-1α release from hMDM following incubation with PAM3CSK4 (1 μg/ml, 3 h) and transfection with LPS (1000 ng/well, 24 h) using LF2000 (0.5 μl/well). Effect of MCC950 (0.37-10 μM) on **(F)** IL-1β and **(G)** IL-1α release from iMGL following incubation with PAM3CSK4 (1 μg/ml, 3 h) and transfection with LPS (1000 ng/well, 24 h) using LF2000 (0.5 μl/well). Data are shown as mean ± S.D. of four technical replicates and representative of three independent experiments for THP-1 and four donors for hMDM. Data are shown as mean ± S.D. of three biological replicates from three donors for iMGL. One‐way ANOVA followed by Tukey’s *post hoc* test. *p < 0.05, **p < 0.01 and ****p < 0.0001.

In addition to NLRC4, the non-canonical caspase-4 inflammasome is active in various CNS disorders including ALS (16, 25, 26). Caspase-4 is predominantly expressed in myeloid cells, T cells and B cells in the periphery and in microglia in the CNS (39, 40). Caspase-4 is a direct sensor of cytosolic LPS and therefore LPS transfection was performed to activate the non-canonical inflammasome pathway (50). A TLR1/2 agonist (PAM3CSK4) has been previously utilized to prime primary innate immune cells prior to a second hit with nigericin or direct cytoplasmic delivery of LPS for canonical and non-canonical stimulation respectively (51). In THP-1, pre-stimulation with PAM3CSK4 followed by LPS transfection triggered a significant increase in IL-1β release (**Figure 7A**). This release was abrogated in NLRP3 KO cells (**Figure 7B**) or following treatment with MCC950 (Emax: 79%, IC_50_: 93 nM; **Figure 7C**). Using the same stimulation paradigm in hMDM, treatment with MCC950 led to a 50-60% decrease IL-1β release following PAM3CSK4 pre-treatment and LPS transfection (**Figure 7D**). IL-1α is a direct substrate for inflammatory caspases of the non-canonical inflammasome pathway (52, 53). In contrast to IL-1β, cytosolic LPS-induced release of IL-1α was unaffected by MCC950 treatment (**Figure 7E**). Unlike THP-1 and hMDM, PAM3CSK4 followed by LPS transfection did not trigger increased release of IL-1β and IL-1α compared to PAM3CSK4 or extracellular LPS stimulation alone in iMGL (**Figures 7F, G**). These data show that NLRP3 inhibition only partially mitigates non-canonical inflammasome activation and with different outcomes in various cell types. This is due in part to differing propensities of cell types such as immortalized lines, primary innate immune, and iPSC-derived microglial cells to respond to non-canonical pathway activating stimuli.

### MCC950 does not inhibit cytokine release and pyroptosis in THP-1 inflammasome GoF mutants

**Figure 8:**
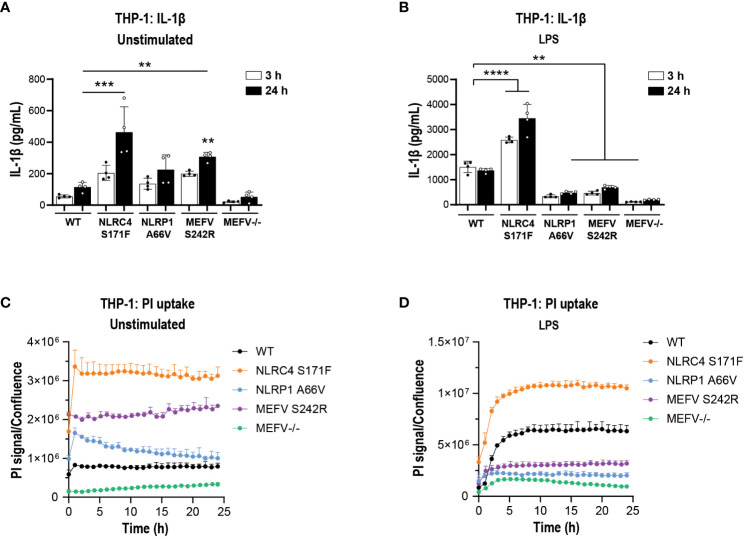
IL-1β secretion and pyroptosis in THP-1 cell lines carrying GoF mutations. PMA-differentiated WT, NLRC4 S171F, NLRP1 A66V, MEFV S242R and MEFV KO THP-1 cells were assessed for the release of IL-1β **(A)** and PI uptake **(C)** in unstimulated condition after 3 h and 24 h. **(B)** PMA-differentiated WT, NLRC4 S171F, NLRP1 A66V, MEFV S242R and MEFV KO THP-1 were pre-incubated with LPS (1 μg/ml, 3 h) and the release of IL-1β **(B)** and PI uptake **(D)** were measured after 3 h and 24 h. Data are shown as mean ± S.D. of four technical replicates from three independent experiments. One‐way ANOVA followed by Tukey’s *post hoc* test. **p < 0.01, ***p < 0.001 and ****p < 0.0001.

Next, we questioned whether MCC950 can block cytokine release and pyroptosis in a system with intrinsically activated inflammasomes. For this we generated gene-edited THP-1 cell lines stably overexpressing GoF mutations in NLRC4, NLRP1 and MEFV genes and an MEFV KO line as a control for pathway inhibition. The specific GoF mutations were selected due to their association with extreme inflammatory phenotypes in patients with autoimmune inflammasomopathies (**Table S1**). Prior to investigating the impact of NLRP3 pathway inhibition, we questioned whether the GoF THP-1 lines produced higher basal levels of IL-1β and whether they would have a propensity for higher cytokine release upon stimulation with LPS. Analysis of culture fluids collected at 3 and 24 h revealed significantly higher IL-1β levels in NLRC4 S171F and MEFV S242R KI lines compared to WT THP-1 (**Figure 8A**). Interestingly, upon LPS stimulation NLRC4 S171F line released significantly higher levels of IL-1β, suggesting an intrinsic propensity for heightened inflammatory response (**Figure 8B**). These results were paralleled by increased pyroptotic activity recorded as a PI uptake signal through time lapse imaging over 24 h. The NLRC4 and MEFV GoF lines displayed higher pyroptosis levels compared to the other lines, with the MEFV line displaying the lowest pyroptotic activity (**Figure 8C**). Interestingly, the NLRC4 line displayed increased sensitivity to LPS stimuli by triggering the highest levels of pyroptosis (**Figure 8D**).

**Figure 9:**
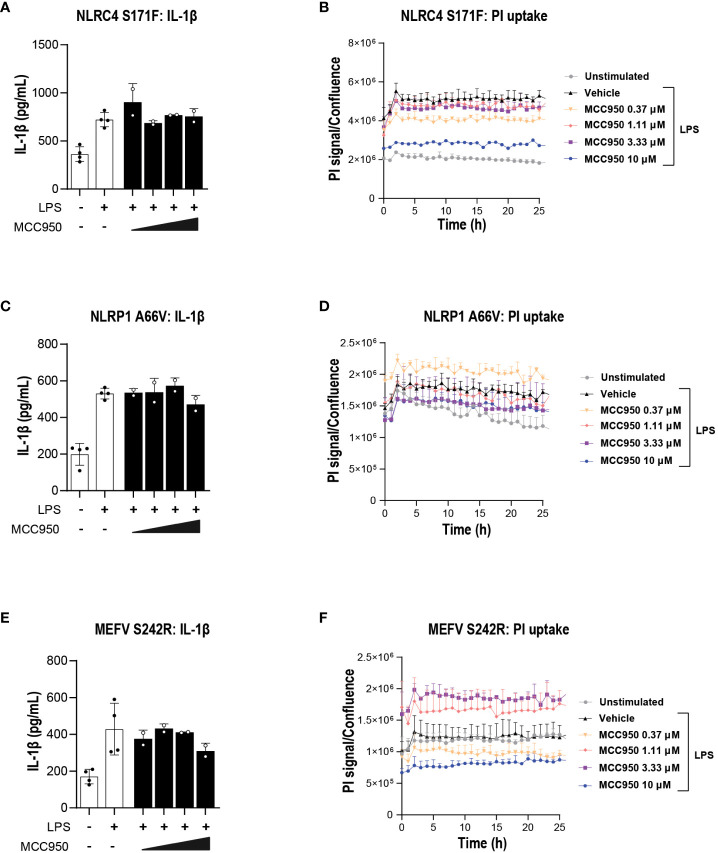
MCC950 does not block inflammasome-mediated pyroptosis in THP-1 carrying inflammasome GoF mutations. Dose response of MCC950 (0.37-10 μM) on IL-1β release in **(A)** NLRC4 S171F, **(C)** NLRP1 A66V, **(E)** MEFV S242R. PI uptake in **(B)** NLRC4 S171F, **(D)** NLRP1 A66V, **(F)** MEFV S242R. MCC950 impact on PI uptake. The dye was added following exposure to increasing concentrations of MCC950 and THP-1 cells were imaged for 25 (h) Data are shown as mean ± S.D. of four technical replicates from three independent experiments.

Having established a suitable assay window in the GoF line, we next tested the impact of MCC950 in cytokine release and pyroptosis. Increasing concentrations of MCC950 did not attenuate IL-1β (**Figures 9A, C, E**) or IL-18 (**Figures S1A–D**) release in the mutant lines. Cytokine findings were paralleled by a lack of activity in MCC950-mediated inhibition of pyroptosis measured by PI uptake following 24 h of LPS stimulation (**Figures 9B, D, F**).

### MCC950 blocks NLRP3- but not NLRC4-induced pyroptosis in healthy and ALS SOD1 mutant iMGL

Since inhibition of NLRP3 in iMGL from healthy controls impacted NLRP3 and in part NLRC4 inflammasome pathway activation, we questioned the impact of NLRP3 inhibition in iMGL generated from ALS patients harboring SOD1 pathogenic mutations. NLRP3 pathway activation and the effects of MCC950 were assessed in three healthy donor and three ALS SOD1 mutant iMGL lines. No apparent differences were observed in the response to LPS and nigericin stimulation between healthy control and SOD1 iMGL. MCC950 treatment strongly decreased IL-1β release across all iMGL lines (**Figures S2A, B**). Similarly, MCC950 attenuated PI uptake in a dose-response manner following NLRP3 inflammasome activation across all control and diseased iMGL lines (**Figures S2C, D**). Next, activation of the NLRC4 inflammasome pathway was investigated *via* Fla-BS transfection as described in iMGL previously. IL-1β release was increased across all iMGL lines following transfection with Fla-BS, with no clear differences in the response observed between healthy control and SOD1 ALS lines (**Figures S2E, F**). While MCC950 treatment inhibited cytosolic Fla-BS-induced IL-1β release by 50-60% across iMGL lines (**Figures S2E, F**), PI uptake was not decreased by MCC950 in both healthy control and SOD1 ALS lines (**Figures S2G, H**). Taken together, these data suggest that MCC950 can block NLRP3 inflammasome and NLRC4 inflammasome-mediated IL-1β release in iMGL from healthy controls and SOD1 ALS patients. On the other hand, NLRP3 inflammasome but not NLRC4 inflammasome-mediated pyroptosis was impacted by NLRP3 inhibition in healthy and SOD1 mutant iMGL.

### Acute systemic administration of MCC950 in a mutant SOD1^G93A^ does not attenuate CNS inflammation

Next, we questioned whether a more complex (*in vivo*) system with multiple triggers of inflammasome activation including protein aggregation, innate immune activation, and progressive neurodegeneration would respond to NLRP3 blockade (54–56). SOD1^G93A^, a transgenic mouse model of familial ALS previously reported to display multiple inflammasome activation in the spinal cord during symptomatic disease, was selected to address the question (57).

Prior to initiating the acute studies and to ensure adequate and sustainable exposure during the acute dosing we evaluated the pharmacokinetic profile of MCC950 in mice at a reportedly efficacious dose of 20 mg/kg, administered *via* oral gavage daily for 7 days (44, 58). The brain-to-plasma ratio (Kp brain) observed for MCC950 in the *in vivo* mouse blood-brain barrier study was 0.005 as previously reported by Gordon et al. (58). Free brain-to-plasma ratio (K_p,uu_ brain) obtained by correcting total concentrations by the fraction unbound (0.0091 and 0.12 for plasma and brain respectively) was 0.06 (**Figures S3A, B**). To estimate target occupancy (TO) of MCC950 in the aimed pharmacology study, PK modeling was performed considering the oral dose of 20 mg/kg, a free brain-to-plasma ratio of 0.06 and an IC_50_ of 7.7 nM based on previous measurements in both mouse bone marrow-derived macrophages (BMDMs) and microglia (44, 58) (**Figures S3C, D**). We estimated average percentage TO values of 81% in plasma and 38% (based on the PK profile from this study) in brain for MCC950 over seven days (**Table S3**, **S4**). Although the estimated average TO for MCC950 over seven days in CNS was not very high, the same dose and route of administration for MCC950 was adopted in the mutant SOD1 model to facilitate comparison with findings from previously published studies (44, 58).

**Figure 10:**
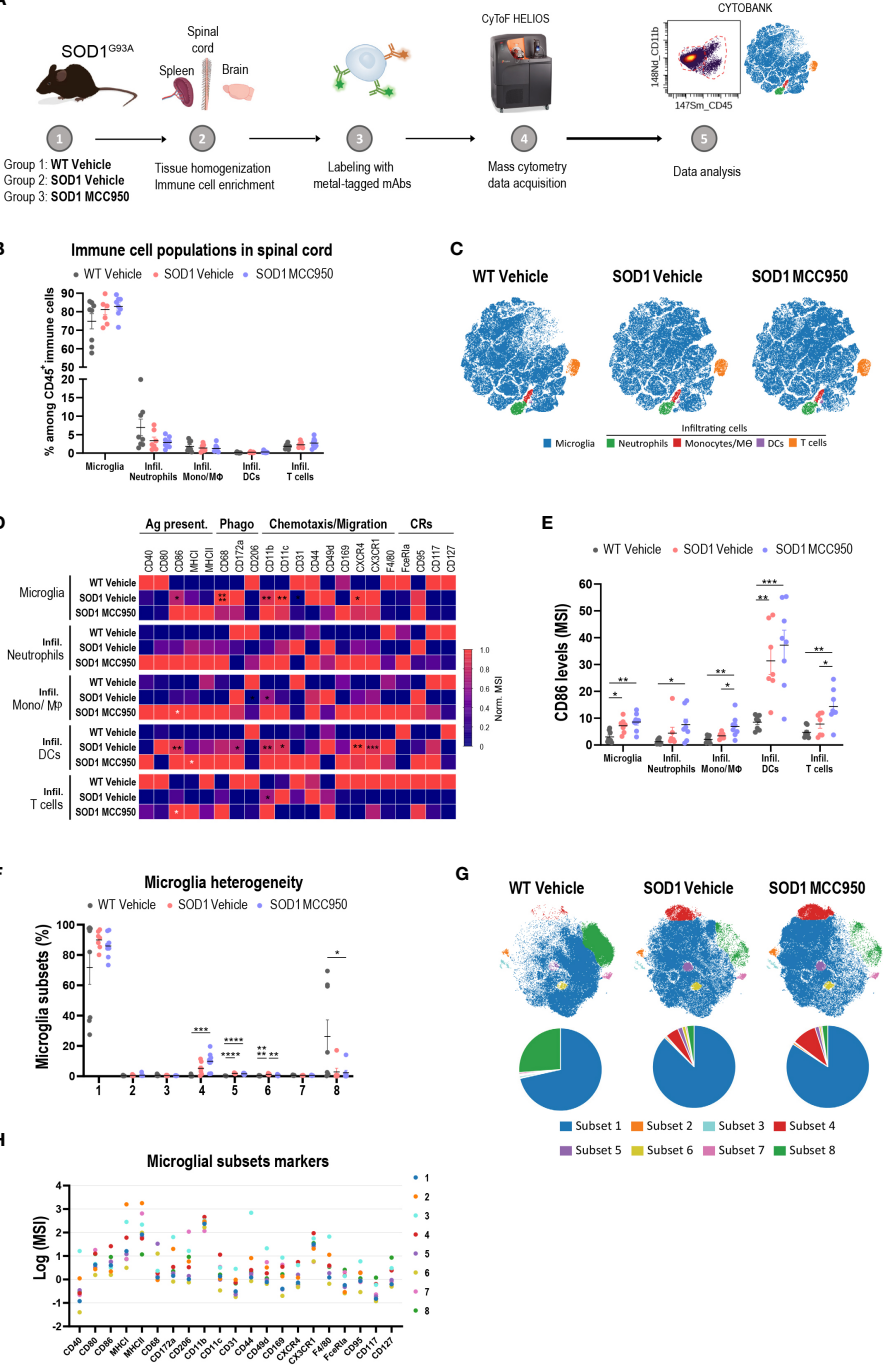
Acute systemic administration of MCC950 in mutant SOD1 mice does not ameliorate spinal cord inflammation. **(A)** Schematic workflow of tissue processing for CyTOF mass cytometry. **(B)** Frequency of resident and infiltrating immune cells in the spinal cord of vehicle-treated WT, vehicle-treated SOD1 and SOD1 mice treated with MCC950. Mono/Mθ: monocytes and macrophages; DCs: dendritic cells. **(C)** Representative two-dimensional projections of single-cell data generated by viSNE of an equal number of CD45^+^ immune cells from each individual animal. Each dot represents one cell. **(D)** Heatmap showing the normalized mean intensity of each marker in the various immune cell subsets. Mean intensity of each marker was normalized according to the maximal and minimal mean intensity value of the marker in each population among the various treatment groups. Ag. Present: antigen presentation; Phago: phagocytosis; CRs: cytokine-related receptors. **(E)** Mean signal intensity levels of CD86 in the different immune cell subsets in the three treatment groups. **(F)** Frequency of microglial subsets 1 to 8 in each sample in the three treatment groups. **(G)** Representative two-dimensional projections of single-cell data generated by viSNE of an equal number of CD45^low^ CX3CR1^+^ microglia from each individual animal. Each dot represents one cell. Pie charts show the relative abundance among microglia of the various subsets. **(H)** Expression levels of functional markers across identified microglial subsets. The dot plot shows the mean (Log2) signal of each marker in each microglial subset. Data in bar graphs shown as mean ± S.E.M. Values for each animal are included (N=8 male mice per treatment group). Statistical analyses were performed using one-way ANOVA with Tukey’s multiple comparisons test. Statistically significant differences between vehicle-treated WT litter mate and SOD1^G93A^ mice are shown in black asterisks. White asterisks represent statistically significant differences in MCC950-treated compared to vehicle-treated SOD1 mice; *p < 0.05, **p < 0.01, ***p < 0.001, ****p < 0.0001.

Next, we investigated the ability of MCC950 to correct inflammation at the cellular level (aberrant microglia phenotypes) beyond correction of proximal NLRP3 pathway inhibition such as cytokine release and pyroptosis. The rationale for employing broader inflammatory readouts was to assess the potential of NLRP3 blockade in a system with complex underlying inflammatory mechanisms beyond NLRP3, including in tandem activation of multiple inflammasomes. In-house longitudinal single cell sequencing studies of SOD1^G93A^ mice at various stages of disease (pre-symptomatic, early symptomatic, peak and end stage) showed a peak signal for NLRP3 and other inflammatory pathways at 100 days of age (data not shown). WT and SOD1 mice were dosed *via* oral gavage with vehicle (PBS) or 20 mg/kg MCC950 for 7 days. Forebrain, spinal cord and spleen were processed for immune phenotyping at single cell resolution by mass cytometry (**Figure 10A**). No significant differences were observed in immune cell population representation and viSNE profiles in the spinal cord of WT mice and SOD1 or vehicle- and MCC950-treated SOD1 mice (**Figures 10B, C**). An in-depth analysis into specific markers selected to provide insights into activation and functional states of the cells, revealed a significant up-regulation of markers linked to phagocytosis (CD68, CD172a), antigen presentation (CD86, MHC-I) and adhesion and movement (CD11b, CD11c, CX3CR1, CXCR4) in microglia and infiltrating immune cells, including monocytes/macrophages, and dendritic cells in SOD1 mice compared to WT controls. Acute administration of MCC950 did not impact the neuroinflammatory profile of mutant SOD1 mouse spinal cord (**Figure 10D**). Only the CD86 was significantly upregulated in infiltrating T cells and neutrophils populations in SOD1 mice with MCC950 treatment (**Figures 10D, E**). Moreover, the expression marker profile was similar in microglial subsets when comparing SOD1 vehicle- and MCC950-treated-mice (**Figures 10F–H**). Deep immune phenotyping of brains and spleens from the same mice did not reveal any remarkable difference between WT- and SOD1-vehicle treated mice nor any significant alterations in numbers or phenotypes of immune populations with NLRP3 inhibition (**Figures S4, S5**). Of note it is not surprising to see a lack of inflammation in the brain of the SOD1 mice considering this is a model characterized by lower motor neuron degeneration and spinal cord inflammation. In summary, these data collectively indicate that acute administration of MCC950 in mutant SOD1 mice did not attenuate spinal cord inflammation.

## Discussion

In recent years the therapeutic potential of NLRP3 inhibition to address various neurodegenerative and demyelinating disorders has gained increasing attention (16, 59). Given the complexity of inflammatory pathways implicated in non-cell autonomous neurodegeneration, we questioned whether blockade of a single inflammasome would sufficiently attenuate inflammatory pathobiology.

To address this question, we employ *in vitro* and *in vivo* models characterized by single and multiple inflammasome pathway activation. *In vitro* we investigate the molecular and cellular underpinnings of inflammasome activation across various innate immune cell populations, shedding light on pathway redundancies and unique responses to canonical and non-canonical inflammasome activation. We show that NLRP3 KO or pharmacological inhibition with MCC950 attenuates cytokine release and pyroptosis when NLRP3 pathway is specifically activated in THP-1, hMonocytes, hMDM and iMGL. In contrast, NLRP3 inhibition results in divergent functional outcomes when NLRC4 pathway is activated in these cells. This is in line with previous findings showing differences in the response of THP-1s and hMDM to MCC950 following flagellin transfection (31). These results could be explained in part by variations in sensitivity to inflammatory stimuli across various cell types. Unlike hMDM, immortalized THP-1 and iMGL display generally blunted inflammatory responses to a range of inflammatory stimuli beyond inflammasomes. Another explanation could be that in the context of inflammasome activation these cells express varying levels of factors that enable preferential execution of select inflammasome pathways. For example, it has been shown that THP-1 lines express lower levels of neuronal/NLR apoptosis inhibitory protein (NAIP) compared to hMDM (31, 60). NAIP physically binds to cytosolic flagellin, interacting with and activating NLRC4 (48). The lower NAIP levels in THP-1 may shift the pathway to compensate through an NLRP3 response to flagellin transfection. Since MCC950 treatment can block NLRC4-mediated IL-1β and IL-18 release but not pyroptosis in iMGL, it is also possible that differences in NAIP levels exist between microglia and peripheral macrophages. This compensatory activity due to lower levels of NAIP, potentially dependent on iMGL differentiation states, could contribute to greater effects of NLRP3 inhibition on NLRC4 activation in these cells (61). Another possible explanation for our findings could be that different flagellins may invoke distinct cellular responses depending on their bacterial source. For example, Fla-BS transfection induced a high inflammatory response in THP-1 and iMGL, whereas hMDM responded best to Fla-ST transfection. Another potential caveat to working with flagellins is signal inflammatory interference through activation of other receptors such as TLR5 expressed in all the cellular systems used in this study. As such a lack of MCC950 efficacy could be explained by activation of inflammatory pathways independent of inflammasomes.

By comparing healthy and SOD1 mutant iMGL, we hypothesized that they may exhibit a heightened immune response to inflammasome stimuli likely due to priming by oxidative stress factors and SOD1 mutant proteins, known triggers of inflammasome activation (23). Surprisingly, responses to NLRP3 or NLRC4 activation, as measured by levels of IL-1β release, did not significantly differ between control and patient lines. One caveat to our experimentation with these lines is a lack of isogenic iPSC controls which were not available for comparison, therefore we cannot exclude the possibility that those differences in amplitude of the immune response between healthy donors and ALS lines may mask any iMGL hyper-responsivity to NLRP3 and/or NLRC4 inflammasome activation. Regarding the non-canonical inflammasome pathway, it is known that caspase-4-associated pyroptosis induces ionic flux which can in turn activate the NLRP3 inflammasome (34–36). In line with previous reports, we found that NLRP3 inhibition can block release of IL-1β in response to LPS transfection in THP-1 and hMDM. Cytosolic LPS-induced IL-1α release was unaffected by NLRP3 inhibition however, as observed previously (44). Why non-canonical inflammasome activators do not induce overt pathway activation in iMGL is unclear. Previous studies suggest lower levels of caspase-4 and caspase-5 in primary microglia may play a role (61). There are several limitations to *in vitro* studies. Firstly, it would have been preferrable to use additional disease-relevant stimuli for multi-inflammasome stimulation; however similar to flagellins, pathogenic aggregates such as amyloid-β and α-synuclein display promiscuity in triggering inflammasome and non-inflammasome pathways alike. As such we had to resort to “cleaner” systems such as THP-1 lines carrying GoF mutations for several inflammasomes, reported in patients suffering from genetic inflammasomopathies. Secondly, PI uptake was used as a read for pyroptosis though it has been widely shown that membrane permeability and PI uptake can be a feature of other types of non-physiological cell death such as necroptosis. Regardless of this caveat PI uptake was used as the most suitable pyroptosis readout in this study, following extensive comparative studies including lactated dehydrogenase (LDH), Gasdermin D, caspase-3/7 measurements in various contexts (data not shown). Despite the limitations, one key learning from this work relates to the differences in functional outcomes between immortalized lines, primary innate and iPSC-derived cells. Although immortalized cell lines can be useful for applications such as HTS screening, our findings indicate that orthogonal validation of hits across cellular systems in the early stages of hit ID and characterization is absolutely critical in the discovery and development of inflammasome and other immune modulators for CNS disorders.

*In vivo* we sought to understand what blockade of NLRP3 pathway and consequently IL-1β release and inflammatory cell death (pyroptosis) would signify for inflammatory pathobiology in a complex system known to display multi-inflammasome activation among other inflammatory mechanisms. To test this hypothesis, we selected the mutant SOD1^G93A^ transgenic mice. While the SOD1^G93A^ model has provided valuable insights into certain pathobiological features of ALS it is burdened by a poor track record of clinical translatability. One likely reason among others is linked to the relevance of SOD1 pathogenic mutations to the broader ALS populations, with SOD1 mutations representing 5% of all familial cases while more than 90% of ALS cases are considered sporadic. Regardless of its limited translatability, the SOD1 model was considered suitable in addressing our question: In a complex system where multiple inflammasomes are activated is blockade of NLRP3 sufficient in blocking inflammation? In-house longitudinal characterization of the model (data not shown) aligned with external findings of increased microglial activation in the spinal cord at pre-symptomatic, disease onset or symptomatic stages depending on the readout sensitivity (62–64). Intervention with MCC950 at day 100 was decided based on peak NLRP3 levels at this timepoint expression being low at pre-symptomatic day 60 and end stages of disease, day 130; data not shown). Using single-cell mass cytometry we identified significant changes in inflammatory markers in resident microglia and infiltrating immune cells in the spinal cord of symptomatic mutant SOD1 mice. While *in vivo* administration of MCC950 has been shown to ameliorate microglial activation in various models of amyloid beta (Aβ), tau and alpha-synuclein (αSyn) pathology (58, 65–67), we find here that acute MCC950 treatment has no visible impact in reversing spinal cord inflammation in mutant SOD1 mice. No impact on microglial populations or levels of activation markers were observed. On the contrary for certain co-stimulatory molecules such as CD86, expression was further increased in the SOD1 mice with MCC950 treatment suggesting exacerbation of certain microglial aberrant behaviors such as antigen-presentation. Interestingly, a recent study in mutant SOD1 mice found that MCC950 administration three times per week from early symptomatic stages until death advanced disease onset and decreased lifespan compared to vehicle-treated controls (21). There could be several reasons explaining these findings. One could be linked to distinct temporal roles of NLRP3 and other inflammasomes in progression of non-cell autonomous neurodegeneration. Another reason may be linked to divergent roles of various receptor families in various neurodegenerative diseases. A long-time assumption has been that neuroinflammation is a common denominator across various diseases. Although as a general statement and depending on one’s definition of neuroinflammation this may be true, we have now deeper molecular and genetic insights into human neurodegenerative disease pathobiology. These insights suggest that not only distinct branches of immunity may be involved in neurodegenerative disease, but also that within the innate immune cell populations certain families of immune receptors can have divergent functions depending on disease and associated pathological cues and disease stages.

In conclusion, our study highlights potential limitations of NLRP3 inhibition in attenuating cytokine release and inflammatory cell death when other inflammasomes are active (**Figure S6**). As such we suggest future therapeutic focus in neurological indications with multi-inflammasome activation should be on converging executer nodes downstream of inflammasomes, rather than single inflammasome-inhibiting approaches.

## Data availability statement

The original contributions presented in the study are included in the article/**Supplementary Material**. Further inquiries can be directed to the corresponding author.

## Ethics statement

The studies involving human participants were reviewed and approved by UCB Biobank and Ethics Committee of UCB Biopharma. The patients/participants provided their written consent to donate the iPSC lines for this study. The animal study was reviewed and approved by Animal Experimentation and Well-Being Ethical Committee compliant with national legislation guidelines (Belgian Royal Decree regarding the protection of laboratory animals of 29 May 2013) and the European directive (2010/63/EU).

## Author contributions

JK and IK conceived and designed the study. M-LC, GG, JV, JL, JG, and AC performed the experiments, analyzed the data, and generated figures. JK, M-LC, and IK wrote the manuscript. All authors contributed to the article and approved the submitted version.

## References

[1] Beers DR Henkel JS Xiao Q Zhao W Wang J Yen AA. Wild-type microglia extend survival in PU.1 knockout mice with familial amyotrophic lateral sclerosis. Proc Natl Acad Sci U.S.A. (2006) 103(43):16021–6. doi: 10.1073/pnas.0607423103 PMC1613228 17043238

[2] Boillée S Yamanaka K Lobsiger CS Copeland NG Jenkins NA Kassiotis G. Onset and progression in inherited ALS determined by motor neurons and microglia. Science (2006) 312(5778):1389–92. doi: 10.1126/science.1123511 16741123

[3] Brettschneider J Libon DJ Toledo JB Xie SX McCluskey L Elman L. Microglial activation and TDP-43 pathology correlate with executive dysfunction in amyotrophic lateral sclerosis. Acta Neuropathol (2012) 123(3):395–407. doi: 10.1007/s00401-011-0932-x 22210083 PMC3595560

[4] Brettschneider J Toledo JB Van Deerlin VM Elman L McCluskey L Lee VM. Microglial activation correlates with disease progression and upper motor neuron clinical symptoms in amyotrophic lateral sclerosis. PloS One (2012) 7(6):e39216. doi: 10.1371/journal.pone.0039216 22720079 PMC3375234

[5] Di Giorgio FP Carrasco MA Siao MC Maniatis T Eggan K. Non-cell autonomous effect of glia on motor neurons in an embryonic stem cell-based ALS model. Nat Neurosci (2007) 10(5):608–14. doi: 10.1038/nn1885 PMC3139463 17435754

[6] Frakes AE Ferraiuolo L Haidet-Phillips AM Schmelzer L Braun L Miranda CJ. Microglia induce motor neuron death *via* the classical NF-κB pathway in amyotrophic lateral sclerosis. Neuron (2014) 81(5):1009–23. doi: 10.1016/j.neuron.2014.01.013 PMC3978641 24607225

[7] Lall D Baloh RH. Microglia and C9orf72 in neuroinflammation and ALS and frontotemporal dementia. J Clin Invest (2017) 127(9):3250–8. doi: 10.1172/JCI90607 PMC5669558 28737506

[8] Yamanaka K Chun SJ Boillee S Fujimori-Tonou N Yamashita H Gutmann DH. Astrocytes as determinants of disease progression in inherited amyotrophic lateral sclerosis. Nat Neurosci (2008) 11(3):251–3. doi: 10.1038/nn2047 PMC3137510 18246065

[9] Chiot A Zaïdi S Iltis C Ribon M Berriat F Schiaffino L. Modifying macrophages at the periphery has the capacity to change microglial reactivity and to extend ALS survival. Nat Neurosci (2020) 23(11):1339–51. doi: 10.1038/s41593-020-00718-z 33077946

[10] Trias E King PH Si Y Kwon Y Varela V Ibarburu S. Mast cells and neutrophils mediate peripheral motor pathway degeneration in ALS. JCI Insight (2018) 3(19). doi: 10.1172/jci.insight.123249 PMC6237484 30282815

[11] Martínez-Muriana A Mancuso R Francos-Quijorna I Olmos-Alonso A Osta R Perry VH. CSF1R blockade slows the progression of amyotrophic lateral sclerosis by reducing microgliosis and invasion of macrophages into peripheral nerves. Sci Rep (2016) 6:25663. doi: 10.1038/srep25663 27174644 PMC4865981

[12] Van Dyke JM Smit-Oistad IM Macrander C Krakora D Meyer MG Suzuki M. Macrophage-mediated inflammation and glial response in the skeletal muscle of a rat model of familial amyotrophic lateral sclerosis (ALS). Exp Neurol (2016) 277:275–82. doi: 10.1016/j.expneurol.2016.01.008 PMC4762214 26775178

[13] Gong T Liu L Jiang W Zhou R. DAMP-sensing receptors in sterile inflammation and inflammatory diseases. Nat Rev Immunol (2020) 20(2):95–112. doi: 10.1038/s41577-019-0215-7 31558839

[14] Platnich JM Muruve DA. NOD-like receptors and inflammasomes: A review of their canonical and non-canonical signaling pathways. Arch Biochem biophys (2019) 670:4–14. doi: 10.1016/j.abb.2019.02.008 30772258

[15] McKenzie BA Dixit VM Power C. Fiery cell death: pyroptosis in the central nervous system. Trends Neurosci (2020) 43(1):55–73. doi: 10.1016/j.tins.2019.11.005 31843293

[16] Voet S Srinivasan S Lamkanfi M van Loo G. Inflammasomes in neuroinflammatory and neurodegenerative diseases. EMBO Mol Med (2019) 11(6). doi: 10.15252/emmm.201810248 PMC6554670 31015277

[17] Johann S Heitzer M Kanagaratnam M Goswami A Rizo T Weis J. NLRP3 inflammasome is expressed by astrocytes in the SOD1 mouse model of ALS and in human sporadic ALS patients. Glia (2015) 63(12):2260–73. doi: 10.1002/glia.22891 26200799

[18] Gugliandolo A Giacoppo S Bramanti P Mazzon E. NLRP3 inflammasome activation in a transgenic amyotrophic lateral sclerosis model. Inflammation (2018) 41(1):93–103. doi: 10.1007/s10753-017-0667-5 28936769

[19] Bellezza I Grottelli S Costanzi E Scarpelli P Pigna E Morozzi G. Peroxynitrite activates the NLRP3 inflammasome cascade in SOD1(G93A) mouse model of amyotrophic lateral sclerosis. Mol Neurobiol (2018) 55(3):2350–61. doi: 10.1007/s12035-017-0502-x 28357805

[20] Lehmann S Esch E Hartmann P Goswami A Nikolin S Weis J. Expression profile of pattern recognition receptors in skeletal muscle of SOD1((G93A)) amyotrophic lateral sclerosis (ALS) mice and sporadic ALS patients. Neuropathol Appl Neurobiol (2018) 44(6):606–27. doi: 10.1111/nan.12483 29575052

[21] Moreno-García L Miana-Mena FJ Moreno-Martínez L de la Torre M Lunetta C Tarlarini C. Inflammasome in ALS skeletal muscle: NLRP3 as a potential biomarker. Int J Mol Sci (2021) 22(5). doi: 10.3390/ijms22052523 PMC7959138 33802349

[22] Meissner F Molawi K Zychlinsky A. Mutant superoxide dismutase 1-induced IL-1beta accelerates ALS pathogenesis. Proc Natl Acad Sci U S A (2010) 107(29):13046–50. doi: 10.1073/pnas.1002396107 PMC2919927 20616033

[23] Deora V Lee JD Albornoz EA McAlary L Jagaraj CJ Robertson AAB. The microglial NLRP3 inflammasome is activated by amyotrophic lateral sclerosis proteins. Glia (2020) 68(2):407–21. doi: 10.1002/glia.23728 31596526

[24] Zhao W Beers DR Bell S Wang J Wen S Baloh RH. TDP-43 activates microglia through NF-κB and NLRP3 inflammasome. Exp Neurol (2015) 273:24–35. doi: 10.1016/j.expneurol.2015.07.019 26222336

[25] Yin P Guo X Yang W Yan S Yang S Zhao T. Caspase-4 mediates cytoplasmic accumulation of TDP-43 in the primate brains. Acta Neuropathol (2019) 137(6):919–37. doi: 10.1007/s00401-019-01979-0 PMC6531422 30810811

[26] Kang SJ Sanchez I Jing N Yuan J. Dissociation between neurodegeneration and caspase-11-mediated activation of caspase-1 and caspase-3 in a mouse model of amyotrophic lateral sclerosis. J Neurosci (2003) 23(13):5455–60. doi: 10.1523/JNEUROSCI.23-13-05455.2003 PMC6741245 12843244

[27] Nigrovic PA Lee PY Hoffman HM. Monogenic autoinflammatory disorders: Conceptual overview, phenotype, and clinical approach. J Allergy Clin Immunol (2020) 146(5):925–37. doi: 10.1016/j.jaci.2020.08.017 PMC7727443 33160483

[28] Albornoz EA Woodruff TM Gordon R. Inflammasomes in CNS diseases. Exp Suppl (2018) 108:41–60. doi: 10.1007/978-3-319-89390-7_3 30536167

[29] Gaidt MM Ebert TS Chauhan D Ramshorn K Pinci F Zuber S. The DNA inflammasome in human myeloid cells is initiated by a STING-cell death program upstream of NLRP3. Cell (2017) 171(5):1110–24.e18. doi: 10.1111/acel.12946 29033128 PMC5901709

[30] Bierschenk D Monteleone M Moghaddas F Baker PJ Masters SL Boucher D. The Salmonella pathogenicity island-2 subverts human NLRP3 and NLRC4 inflammasome responses. J Leukoc Biol (2019) 105(2):401–10. doi: 10.1002/JLB.MA0318-112RR 30368901

[31] Gram AM Wright JA Pickering RJ Lam NL Booty LM Webster SJ. Salmonella flagellin activates NAIP/NLRC4 and canonical NLRP3 inflammasomes in human macrophages. J Immunol (2021) 206(3):631–40. doi: 10.4049/jimmunol.2000382 PMC7812056 33380493

[32] Qu Y Misaghi S Newton K Maltzman A Izrael-Tomasevic A Arnott D. NLRP3 recruitment by NLRC4 during Salmonella infection. J Exp Med (2016) 213(6):877–85. doi: 10.1084/jem.20132234 PMC4886354 27139490

[33] Broz P Newton K Lamkanfi M Mariathasan S Dixit VM Monack DM. Redundant roles for inflammasome receptors NLRP3 and NLRC4 in host defense against Salmonella. J Exp Med (2010) 207(8):1745–55. doi: 10.1084/jem.20100257 PMC2916133 20603313

[34] Baker PJ Boucher D Bierschenk D Tebartz C Whitney PG D’Silva DB. NLRP3 inflammasome activation downstream of cytoplasmic LPS recognition by both caspase-4 and caspase-5. Eur J Immunol (2015) 45(10):2918–26. doi: 10.1002/eji.201545655 26173988

[35] Schmid-Burgk JL Gaidt MM Schmidt T Ebert TS Bartok E Hornung V. Caspase-4 mediates non-canonical activation of the NLRP3 inflammasome in human myeloid cells. Eur J Immunol (2015) 45(10):2911–7. doi: 10.1002/eji.201545523 26174085

[36] Rühl S Broz P. Caspase-11 activates a canonical NLRP3 inflammasome by promoting K(+) efflux. Eur J Immunol (2015) 45(10):2927–36. doi: 10.1002/eji.201545772 26173909

[37] Abud EM Ramirez RN Martinez ES Healy LM Nguyen CHH Newman SA. iPSC-derived human microglia-like cells to study neurological diseases. Neuron (2017) 94(2):278–93 e9. doi: 10.1016/j.neuron.2017.03.042 28426964 PMC5482419

[38] Guarda G Zenger M Yazdi AS Schroder K Ferrero I Menu P. Differential expression of NLRP3 among hematopoietic cells. J Immunol (2011) 186(4):2529–34. doi: 10.4049/jimmunol.1002720 21257968

[39] Uhlén M Fagerberg L Hallström BM Lindskog C Oksvold P Mardinoglu A. Proteomics. Tissue-based map of the human proteome. Science (2015) 347(6220):1260419. doi: 10.1126/science.126041 25613900

[40] The Human Protein Atlas . Available at: http://www.proteinatlas.org/.

[41] Zhang Y Sloan SA Clarke LE Caneda C Plaza CA Blumenthal PD. Purification and characterization of progenitor and mature human astrocytes reveals transcriptional and functional differences with mouse. Neuron (2016) 89(1):37–53. doi: 10.1016/j.neuron.2015.11.013 26687838 PMC4707064

[42] Brain RNA-Seq . Available at: https://www.brainrnaseq.org/.

[43] Yang Y Wang H Kouadir M Song H Shi F. Recent advances in the mechanisms of NLRP3 inflammasome activation and its inhibitors. Cell Death Dis (2019) 10(2):128. doi: 10.1038/s41419-019-1413-8 30755589 PMC6372664

[44] Coll RC Robertson AA Chae JJ Higgins SC Muñoz-Planillo R Inserra MC. A small-molecule inhibitor of the NLRP3 inflammasome for the treatment of inflammatory diseases. Nat Med (2015) 21(3):248–55. doi: 10.1038/nm.3806 PMC4392179 25686105

[45] Primiano MJ Lefker BA Bowman MR Bree AG Hubeau C Bonin PD. Efficacy and pharmacology of the NLRP3 inflammasome inhibitor CP-456,773 (CRID3) in murine models of dermal and pulmonary inflammation. J Immunol (2016) 197(6):2421–33. doi: 10.4049/jimmunol.1600035 27521339

[46] Rui W Xiao H Fan Y Ma Z Xiao M Li S. Systemic inflammasome activation and pyroptosis associate with the progression of amnestic mild cognitive impairment and Alzheimer’s disease. J Neuroinflammation (2021) 18(1):280. 34856990 10.1186/s12974-021-02329-2 PMC8638109

[47] Freeman L Guo H David CN Brickey WJ Jha S Ting JP. NLR members NLRC4 and NLRP3 mediate sterile inflammasome activation in microglia and astrocytes. J Exp Med (2017) 214(5):1351–70. doi: 10.1084/jem.20150237 PMC5413320 28404595

[48] Duncan JA Canna SW. The NLRC4 inflammasome. Immunol Rev (2018) 281(1):115–23. doi: 10.1111/imr.12607 PMC5897049 29247997

[49] Felderhoff-Mueser U Schmidt OI Oberholzer A Buhrer C Stahel PF. IL-18: a key player in neuroinflammation and neurodegeneration? Trends Neurosci (2005) 28(9):487–93. doi: 10.1016/j.tins.2005.06.008 16023742

[50] Matikainen S Nyman TA Cypryk W. Function and regulation of noncanonical caspase-4/5/11 inflammasome. J Immunol (2020) 204(12):3063–9. doi: 10.4049/jimmunol.2000373 32513874

[51] Gaidt MM Ebert TS Chauhan D Ramshorn K Pinci F Zuber S. The DNA inflammasome in human myeloid cells is initiated by a STING-cell death program upstream of NLRP3. Cell (2017) 171(5):1110–24 e18. doi: 10.1016/j.cell.2017.09.039 29033128 PMC5901709

[52] Wiggins KA Parry AJ Cassidy LD Humphry M Webster SJ Goodall JC. IL-1α cleavage by inflammatory caspases of the noncanonical inflammasome controls the senescence-associated secretory phenotype. Aging Cell (2019) 18(3):e12946. 30916891 10.1111/acel.12946 PMC6516163

[53] Kelley N Jeltema D Duan Y He Y. The NLRP3 inflammasome: an overview of mechanisms of activation and regulation. Int J Mol Sci (2019) 20(13). doi: 10.3390/ijms20133328 PMC6651423 31284572

[54] Stieber A Gonatas JO Gonatas NK. Aggregates of mutant protein appear progressively in dendrites, in periaxonal processes of oligodendrocytes, and in neuronal and astrocytic perikarya of mice expressing the SOD1G93A mutation of familial amyotrophic lateral sclerosis. J Neurological Sci (2000) 177(2):114–23. doi: 10.1016/S0022-510X(00)00351-8 10980307

[55] Butovsky O Jedrychowski MP Cialic R Krasemann S Murugaiyan G Fanek Z. Targeting miR-155 restores abnormal microglia and attenuates disease in SOD1 mice. Ann Neurol (2015) 77(1):75–99. doi: 10.1002/ana.24304 25381879 PMC4432483

[56] Heiman-Patterson TD Deitch JS Blankenhorn EP Erwin KL Perreault MJ Alexander BK. Background and gender effects on survival in the TgN(SOD1-G93A)1Gur mouse model of ALS. J Neurol Sci (2005) 236(1-2):1–7. doi: 10.1016/j.jns.2005.02.006 16024047

[57] Hummel C Leylamian O Posch A Weis J Aronica E Beyer C. Expression and Cell Type-specific Localization of Inflammasome Sensors in the Spinal Cord of SOD1((G93A)) Mice and Sporadic Amyotrophic lateral sclerosis Patients. Neuroscience (2021) 463:288–302. doi: 10.1016/j.neuroscience.2021.03.023 33781799

[58] Gordon R Albornoz EA Christie DC Langley MR Kumar V Mantovani S. Inflammasome inhibition prevents α-synuclein pathology and dopaminergic neurodegeneration in mice. Sci Transl Med (2018) 10(465). doi: 10.1126/scitranslmed.aah4066 PMC6483075 30381407

[59] Heneka MT McManus RM Latz E. Inflammasome signalling in brain function and neurodegenerative disease. Nat Rev Neurosci (2018) 19(10):610–21. doi: 10.1038/s41583-018-0055-7 30206330

[60] Kortmann J Brubaker SW Monack DM. Cutting edge: inflammasome activation in primary human macrophages is dependent on flagellin. J Immunol (2015) 195(3):815–9. doi: 10.4049/jimmunol.1403100 PMC4505955 26109648

[61] Burm SM Zuiderwijk-Sick EA t Jong AE van der Putten C Veth J Kondova I. Inflammasome-induced IL-1β secretion in microglia is characterized by delayed kinetics and is only partially dependent on inflammatory caspases. J Neurosci (2015) 35(2):678–87. doi: 10.1523/JNEUROSCI.2510-14.2015 PMC6605364 25589762

[62] Alexianu ME Kozovska M Appel SH. Immune reactivity in a mouse model of familial ALS correlates with disease progression. Neurology (2001) 57(7):1282–9. doi: 10.1212/WNL.57.7.1282 11591849

[63] Maniatis S Äijö T Vickovic S Braine C Kang K Mollbrink A. Spatiotemporal dynamics of molecular pathology in amyotrophic lateral sclerosis. Science (2019) 364(6435):89–93. doi: 10.1126/science.aav9776 30948552

[64] Lewis KE Rasmussen AL Bennett W King A West AK Chung RS. Microglia and motor neurons during disease progression in the SOD1G93A mouse model of amyotrophic lateral sclerosis: changes in arginase1 and inducible nitric oxide synthase. J Neuroinflammation (2014) 11:55. doi: 10.1186/1742-2094-11-55 24655927 PMC3994340

[65] Stancu IC Cremers N Vanrusselt H Couturier J Vanoosthuyse A Kessels S. Aggregated Tau activates NLRP3-ASC inflammasome exacerbating exogenously seeded and non-exogenously seeded Tau pathology in vivo. Acta Neuropathol (2019) 137(4):599–617. doi: 10.1007/s00401-018-01957-y 30721409 PMC6426830

[66] Dempsey C Rubio Araiz A Bryson KJ Finucane O Larkin C Mills EL. Inhibiting the NLRP3 inflammasome with MCC950 promotes non-phlogistic clearance of amyloid-β and cognitive function in APP/PS1 mice. Brain behavior immunity (2017) 61:306–16. doi: 10.1016/j.bbi.2016.12.014 28003153

[67] Lonnemann N Hosseini S Marchetti C Skouras DB Stefanoni D D’Alessandro A. The NLRP3 inflammasome inhibitor OLT1177 rescues cognitive impairment in a mouse model of Alzheimer’s disease. Proc Natl Acad Sci U S A (2020) 117(50):32145–54. doi: 10.1073/pnas.2009680117 PMC7749353 33257576

