# Supplementary material for: Divergent functional outcomes of NLRP3 blockade downstream of multi-inflammasome activation: therapeutic implications for ALS
Source: Front Immunol. 2023 Jul 27;14:1190219. doi: 10.3389/fimmu.2023.1190219 (PMC10415077; doi:10.3389/fimmu.2023.1190219)
Supplement: Supplementary file 1 [file DataSheet_1.docx]

Supplementary Material

##
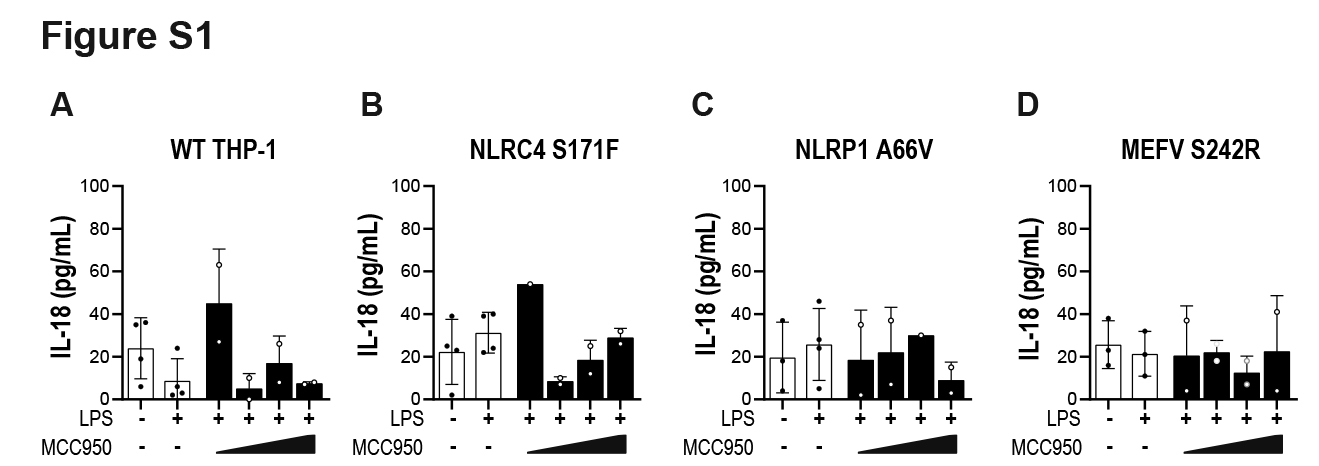


**Supplementary Figure 1:** **MCC950 does not significantly impact inflammasome-mediated IL-18 release in THP-1 inflammasome mutant lines.** PMA-differentiated WT, NLRC4 S171F, NLRP1 A66V, MEFV S242R and MEFV KO THP-1 cells were pre-incubated with LPS (1 μg/ml, 3 h) and assessed for the release of IL-18 after 3 h. Effects of MCC-950 (0.37-10 μM) on IL-18 release (3 h) from **(A)** WT, **(B)** NLRC4 S171F, **(C)** NLRP1 A66V, **(D)** MEFV S242R following LPS stimulation. Data are mean ± S.D. of three technical replicates from three independent experiments.

**
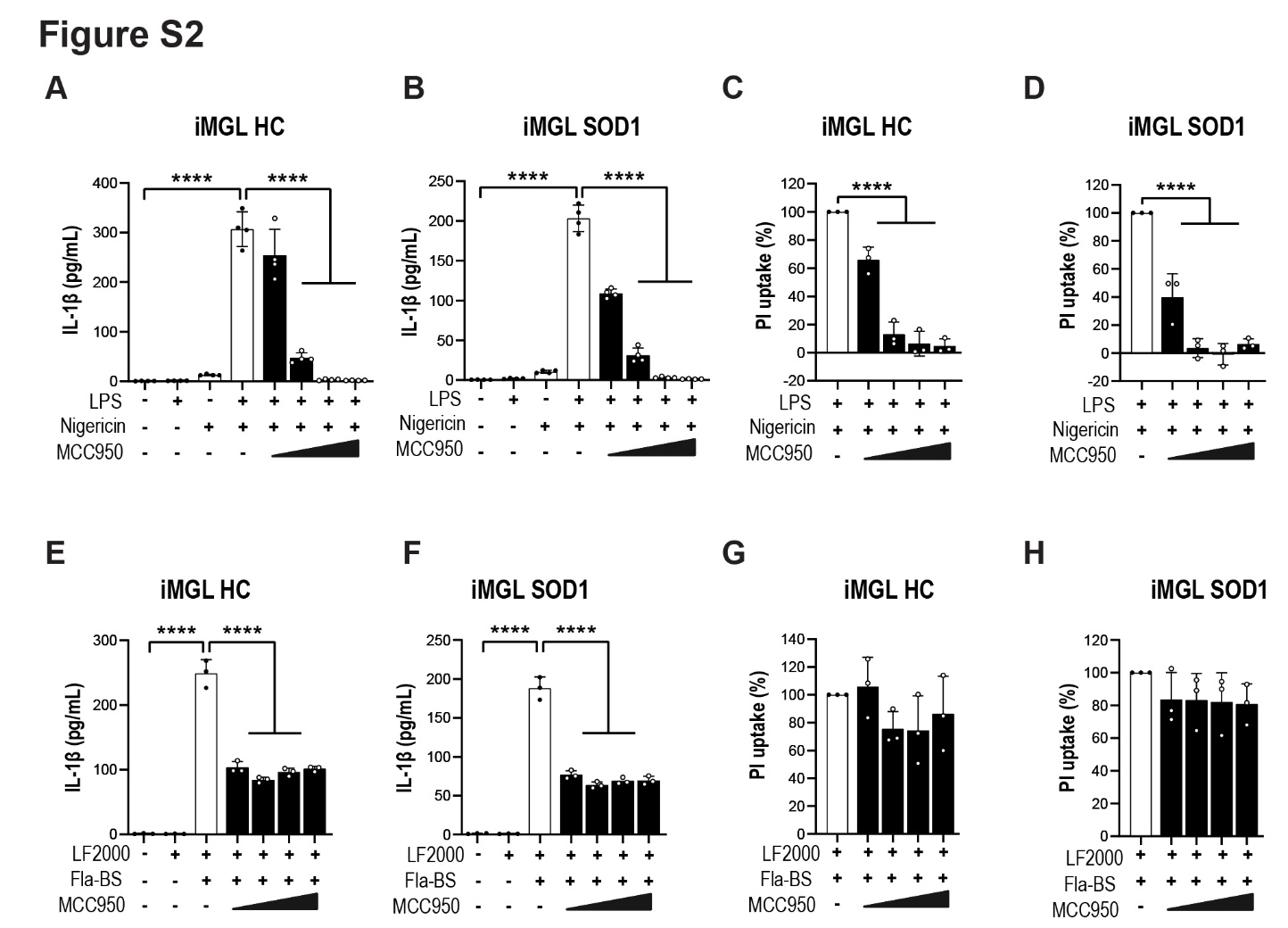

Supplementary Figure 2: MCC950 blocks NLRP3- but not NLRC4- inflammasome-mediated pyroptosis in iMGL from healthy controls (HC) and SOD1-ALS patients.** Effects of MCC-950 (0.37-10 μM) on **(A, B)** IL-1β release and **(C, D)** PI uptake in healthy control and SOD1 mutant iMGL, following NLRP3 inflammasome activation (via LPS and nigericin stimulation). IL-1β levels and PI uptake was assessed 3 h after nigericin exposure. Effects of MCC-950 (0.37-10 μM) on **(E, F)** IL-1β release and **(G, H)** PI uptake in HC and SOD1mutant iMGL following NLRC4 inflammasome activation (via Fla-BS transfection). IL-1β levels and PI uptake was assessed 24 h after flagellin transfection. Data are mean ± S.D. of three technical replicates from three human donors per genotype (HC and SOD1). One‐way ANOVA followed by Tukey’s post hoc test. *****p* < 0.0001.

**
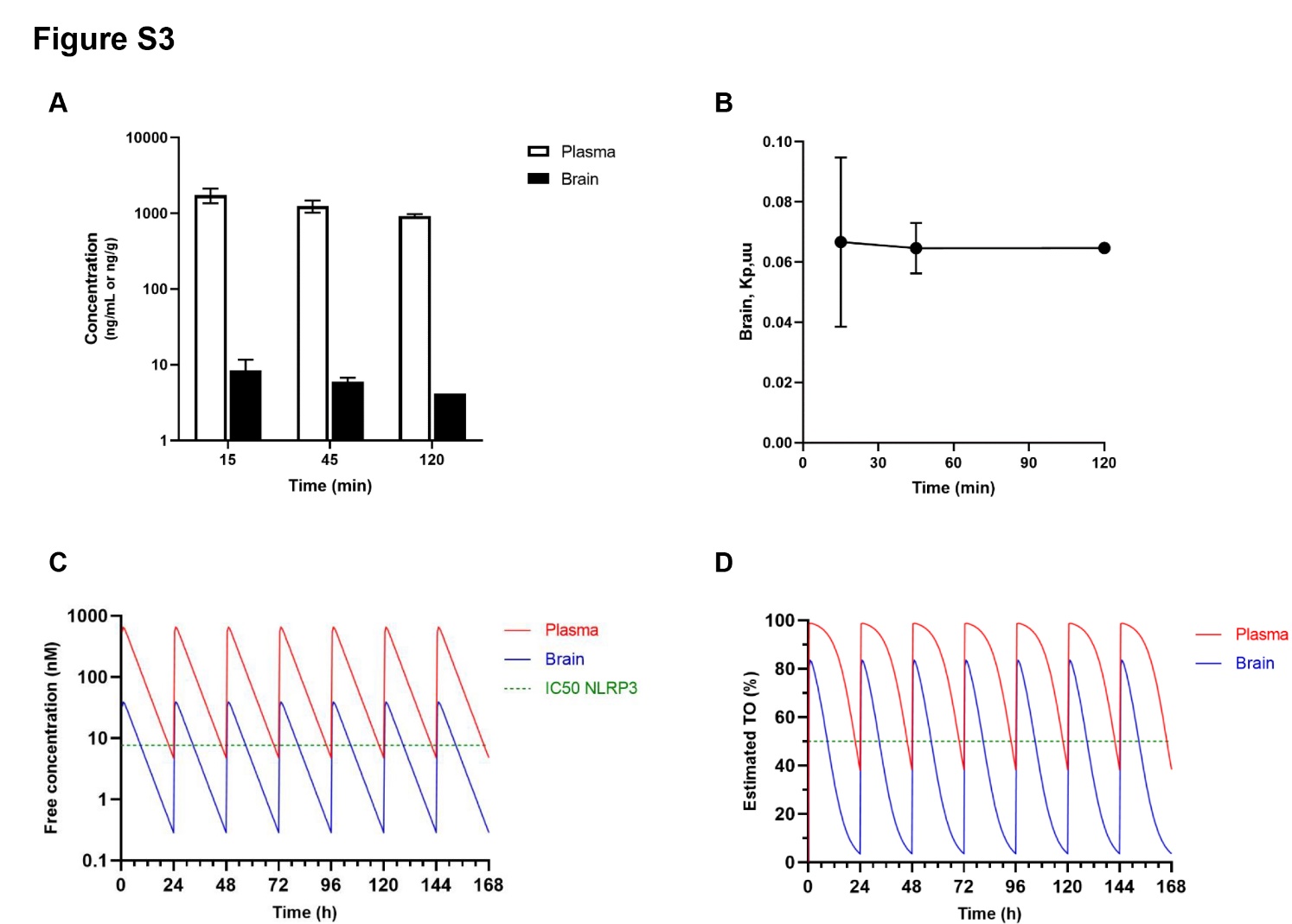
**

**Supplementary Figure 3: (A)** MCC-950 concentration in mouse plasma and perfused brain following intravenous administration (0.5 mg/kg; n = 3 mice per time point). **(B).** Free brain-to-plasma concentration ratio (Kp_,uu brain_) of MCC-950 observed in mice following the intravenous administration of a dose of 0.5mg/kg and showing a fast plasma-brain equilibrium and a brain Kp_,uu_ of 0.06. (**C)**. Predicted free plasma and brain concentrations in mice following oral administration of MCC-950 at a dose of 20mg/kg/day for 7 days. Profiles were predicted from a mono-compartmental model (ka: 2.5h^-1^, Cl/F: 120mL∙h^-1^∙kg^-1^, V/F: 550mL∙kg^-1^) and transformation in free concentrations after correction by unbound fraction and brain-to-plasma ratio. **(D)** Predicted levels of target occupancy (%TO) in plasma and brain in mice administered orally with MCC-950 at a dose of 20mg/kg/day for 7 days.

**
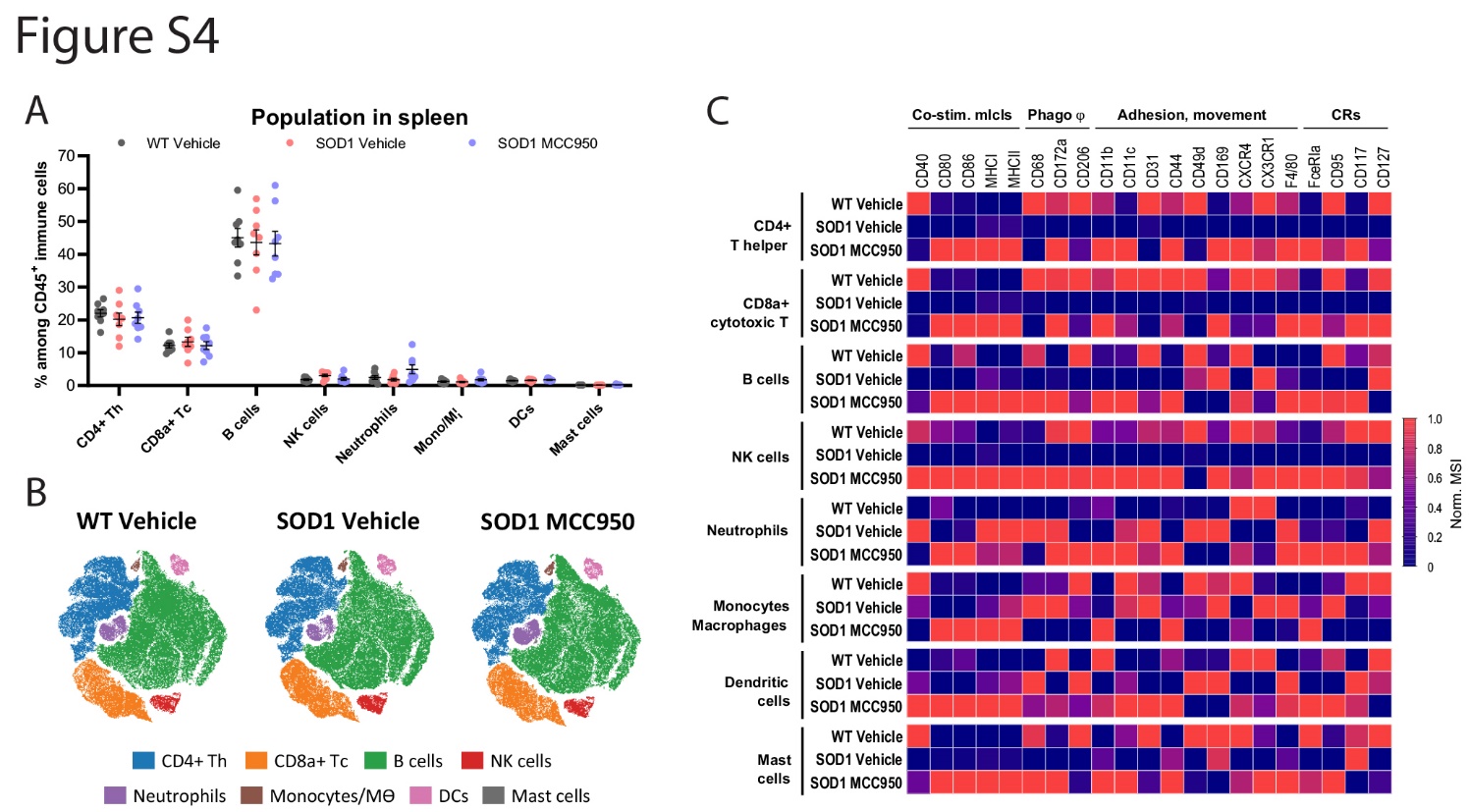
**

**Supplementary Figure 4: Impact of MCC950 acute systemic dosing in mutant SOD1 mice peripheral immunity (spleens). (A)** Frequency of resident and infiltrating immune cells in the spleen of vehicle-treated wild type, vehicle-treated SOD1 and SOD1 mice treated with MCC-950. Th: T helper cells, Tc: cytotoxic T cells, DCs: dendritic cells. **(B)** Representative two-dimensional projections of single-cell data generated by viSNE of an equal number of CD45^+^ immune cells from each individual animal. Each dot represents one cell. **(C)** Heatmap showing the normalized mean intensity of each marker in the various immune cell subsets. Mean intensity of each marker was normalized according to the maximal and minimal mean intensity value of the marker in each population among the various treatment groups. Data in graphs is captured as mean ± S.E.M from eight animals per treatment group. Statistical analyses used one-way ANOVA with Tukey’s multiple comparisons test with p <0.05 considered significant.

**
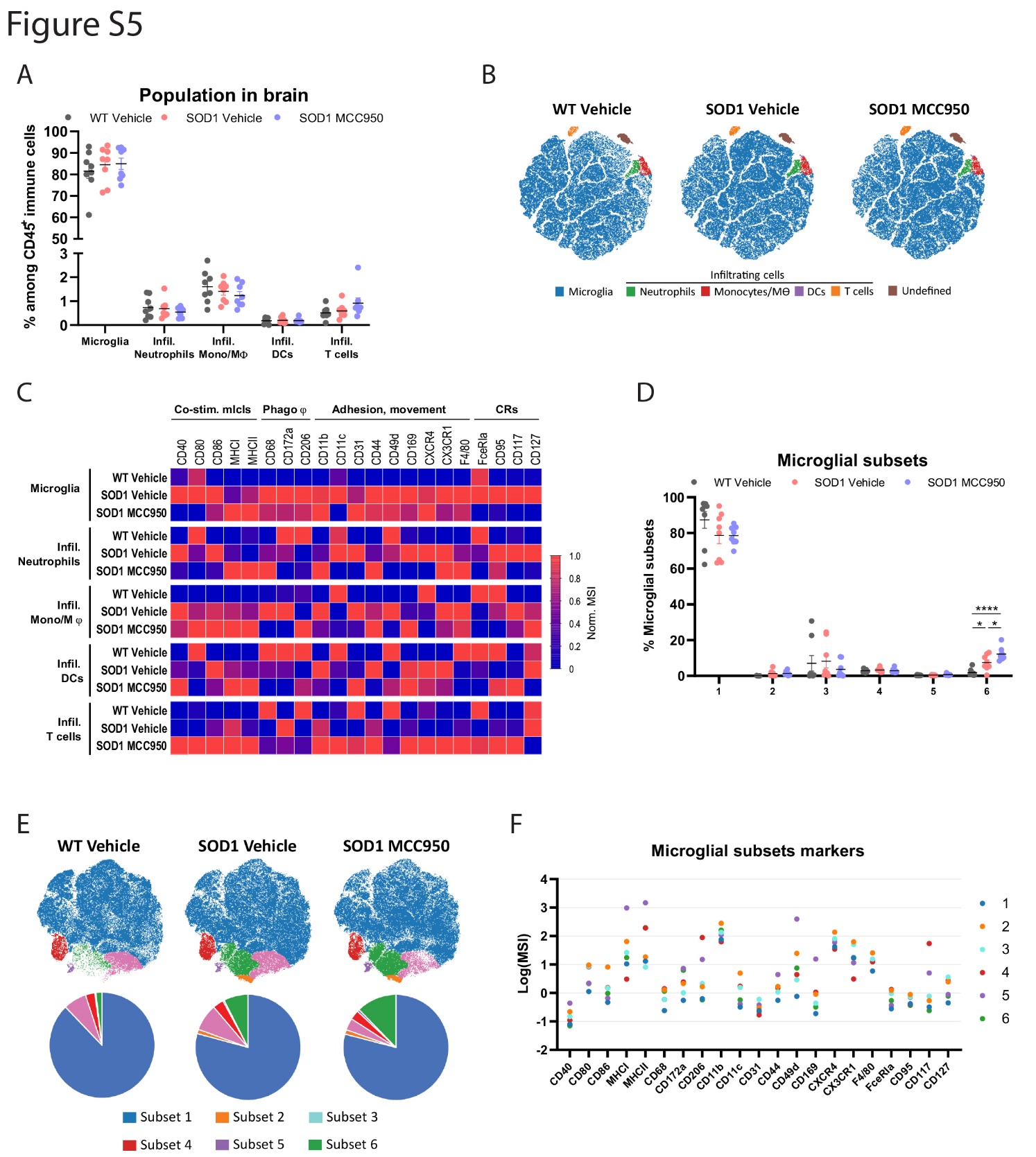
**

**Supplementary Figure 5: Impact of MCC950 acute systemic dosing in mutant SOD1 mice brains. (A)** Frequency of resident and infiltrating immune cells in the brain of vehicle-treated wild type, vehicle-treated SOD1 and SOD1 mice treated with MCC-950. Mono/Mϴ: monocytes and macrophages; DCs: dendritic cells. **(B)** Representative two-dimensional projections of single-cell data generated by viSNE of an equal number of CD45^+^ immune cells from each individual animal. Each dot represents one cell. **(C)** Heatmap showing the normalized mean intensity of each marker in the various immune cell subsets. Mean intensity of each marker was normalized according to the maximal and minimal mean intensity value of the marker in each population among the various treatment groups. Crs: cytokine-related receptors. **(D)** Frequency of microglial subsets 1 to 6 in each sample in the three treatment groups. **(E)** Representative two-dimensional projections of single-cell data generated by viSNE of an equal number of CD45^low^ CX3CR1^+^ microglia from each individual animal. Each dot represents one cell. Pie charts below show the relative abundance among microglia of the various subsets. **(F)** The dot plot shows mean (Log2) signal of each marker in each microglial subset. Data in graphs is captured as mean ± S.E.M from eight animals per treatment group. Statistical analyses performed as one-way ANOVA with Tukey’s multiple comparisons test with p <0.05 considered significant.

**Figure S6**

**
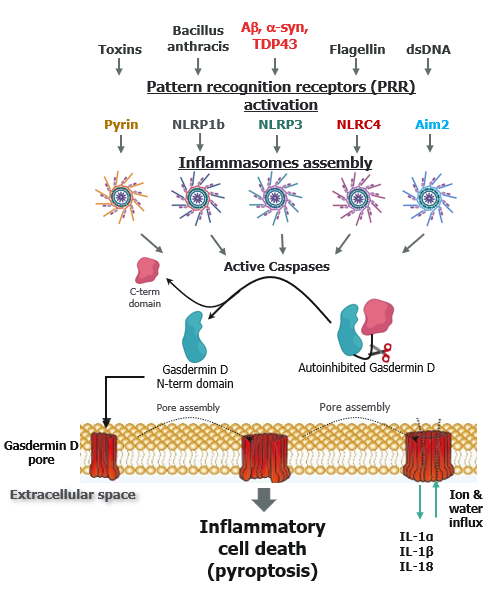
**

**Figure S6.** Activation of several inflammasomes and pyroptotic inflammation is a common denominator across neurodegenerative proteinopathies including AD, PD and ALS. Inhibition of a single inflammasome such as NLRP3 does not mitigate pyroptosis-induced inflammation in specific innate immune cells when other inflammasomes are activated. Future therapeutic focus in neurological indications with multi-inflammasome activation should be on converging executer nodes downstream of inflammasomes rather than single inflammasome-inhibiting approaches.

## Supplementary Tables

**Supplementary Table 1: THP-1 cell lines carrying GoF mutations for various inflammasomopathies**

| **Gene Edited** | **Mutation** | **Modification** | **Zygosity** | **Cell Type** | **Associated Pathology** |
| --- | --- | --- | --- | --- | --- |
| NLRP1 | A66V (GCC>GtC) | knock-in | homozygous | THP-1 | Multiple Self-Healing Palmoplantar Carcinoma  (MSPC) |
| NLRC4 | S171F (TCT>TtT) | knock-in | homozygous | THP-1 | Autoinflammation and infantile Enterocolitis (AIFEC) |
| MEFV | S242R (AGC>AGg) | knock-in | homozygous | THP-1 | Pyrin-Associated Autoinflammation with Neutrophilic Dermatosis (PAAND) |
| MEFV | n/a | knockout | homozygous | THP-1 | n/a |
| NLRP3 | n/a | knockout | homozygous | THP-1 | n/a |

n/a, not applicable

**Supplementary Table 2: Human healthy and ALS patient iPSC lines**

| **iPSC Line Name** | **Source** | **Disease** | **Mutation** | **Parent Cell Type** |
| --- | --- | --- | --- | --- |
| HC 1 | Cedars-Sinai iPSC core | Healthy | n/a | PBMC |
| HC 2 | Cedars-Sinai iPSC core | Healthy | n/a | PBMC |
| HC 3 | Cedars-Sinai iPSC core | Healthy | n/a | PBMC |
| SOD1-ALS 1 | Cedars-Sinai iPSC core | ALS | SOD1 / A4V | PBMC |
| SOD1-ALS 2 | Cedars-Sinai iPSC core | ALS | SOD1 / I114T | PBMC |
| SOD1-ALS 3 | Cedars-Sinai iPSC core | ALS | SOD1 / Unknown | PBMC |
|  |  |  |  |  |

**Supplementary Table 3: Estimated free concentration and Target Occupancy (TO) in plasma and brain at steady state**

| **Variable** | **Units** | **Average** | **Median** | **Maximum** | **Minimum** |
| --- | --- | --- | --- | --- | --- |
| Free plasma concentration | nM | 155 | 62.3 | 651 | 4.8 |
| Free brain concentration | nM | 9.3 | 3.7 | 39.1 | 0.29 |
| Estimated TO in plasma | % | 81.2 | 89.0 | 98.8 | 38.4 |
| Estimated TO in brain | % | 38.0 | 32.7 | 83.5 | 3.60 |

**Supplementary Table 4: PK/PD parameters used for the estimation of exposure and target occupancy**

| **PK Parameters model** | **Units** | **Estimate** |
| --- | --- | --- |
| Ka | h^-1^ | 2.5 |
| Cl/F | mL*h^-1^*kg^-1^ | 120 |
| V/F | mL*kg^-1^ | 550 |
| Brain/Plasma Kp_, uu_ | - | 0.06 |
| NLRP3 IC50 | nM | 7.7 |

**Supplementary Table 5: Mass cytometry (CyToF) antibody panel**

| **UniProt** | **IsotopeTag** | **Antigen** | **Lineage/Function** | **Supplier** | **Catalog No.** |
| --- | --- | --- | --- | --- | --- |
| P35461 | 141Pr | Ly-6G | Neutrophils | Fluidigm | 3141008B |
| P97797 | 142Nd | CD172a | Phagocytosis | BioLegend | 144002 |
| P01899 | 144Nd | MHC-I | Antigen presentation | Fluidigm | 3144016B |
| P06332 | 145Nd | CD4 | T helper cells | Fluidigm | 3145002B |
| Q61549 | 146Nd | F4/80 | Macrophages, microglia; adhesion | Fluidigm | 3146008B |
| P06800 | 147Sm | CD45 | Pan-immune | Fluidigm | 3147003B |
| P05555 | 148Nd | CD11b | Macrophages, microglia | Fluidigm | 3148003B |
| P31996 | 149Sm | CD68 | Phagocytosis | BioLegend | 137002 |
| P15379 | 150Nd | CD44 | Adhesion & movement | Fluidigm | 3150018B |
| Q00651 | 151Eu | CD49d | Adhesion | Fluidigm | 3151016B |
| P22646 | 152Sm | CD3e | T cells | Fluidigm | 3152004B |
| P01731 | 153Eu | CD8a | Cytotoxic T cells | Fluidigm | 3153012B |
| Q9ES52 | 154Sm | TER119 | Red blood cells | Fluidigm | 3154005B |
| P70658 | 159Tb | CXCR4 | Adhesion & movement | Fluidigm | 3159030B |
| P27512 | 161Dy | CD40 | Co-stimulatory molecule | Fluidigm | 3161020B |
| Q9QXH4 | 162Dy | CD11c | Dendritic cells; microglial activation | Fluidigm | 3162017B |
| P25446 | 163Dy | CD95 (FasR) | Death receptor | BioLegend | 152602 |
| Q9Z0D9 | 164Dy | CX3CR1 | Microglia | Fluidigm | 3164023B |
| Q08481 | 165Ho | CD31 | Endothelial cells; adhesion | Fluidigm | 3165013B |
| P25918 | 166Er | CD19 | B cells | Fluidigm | 3166015B |
| Q8C567 | 167Er | CD335 | NK cells | Fluidigm | 3167008B |
| P09581 | 168Er | CSF1R/CD115 | Microglia, monocyte/macrophage differentiation | BioLegend | 135521 |
| Q2HZ94 | 169Tm | CD206/MMR | PRR | Fluidigm | 3169021B |
| Q62230 | 170Er | CD169 | Perivascular macrophages | Fluidigm | 3170018B |
| Q00609 | 171Yb | CD80 | Co-stimulatory molecule | Fluidigm | 3171008B |
| P42082 | 172Yb | CD86 | Co-stimulatory molecule | Fluidigm | 3172016B |
| P05532 | 173Yb | CD117 (c-kit) | Cytokine receptor | Fluidigm | 3173004B |
| Q61790 | 174Yb | CD223 (Lag3) | Antigen presentation | Fluidigm | 3174019B |
| Q60943 | 175Lu | CD127/IL7Ra | Cytokine receptor | Fluidigm | 3175006B |
| P20489 | 176Yb | FceRIa | IgE Fc receptor, mast cells | Fluidigm | 3176006B |
| P04441 | 209Bi | I-A/I-E | Antigen presentation | Fluidigm | 3209006B |
